# Targeting NDUFS8 in basal forebrain ameliorates cognitive decline related to chronic cerebral hypoperfusion

**DOI:** 10.7150/thno.117635

**Published:** 2026-01-01

**Authors:** Yang Qu, Xiaoting Xu, Xuqiao Wang, Jinan Yang, Liye Zhang, Tao Wang, Xin Tang, Wei Cheng, Jing Li, Jing Ma, Yan Wu, Wentao Xu, Qin Wang, Lu Zeng, Xiaobin An, Dongyang Wang, Meijie Chen, Guitian Cong, Yonghui Wu, Bin Sun, Jing Ai

**Affiliations:** 1 Department of Pharmacology, College of Pharmacy of Harbin Medical University, Harbin 150081, China.; 2 State Key Laboratory of Frigid Zone Cardiovascular Diseases (SKLFZCD), College of Pharmacy of Harbin Medical University, Harbin 15008l, China.; 3 Research Center for Pharmacoinformatic, College of Pharmacy of Harbin Medical University, Harbin 150081, China.; 4 Institute of Basic Medical Sciences, Chinese Academy of Medical Sciences, Department of Human Anatomy, Histology and Embryology, Neuroscience Center, Joint Laboratory of Anesthesia and Pain, School of Basic Medicine, Peking Union Medical College, Beijing 100006, China.; 5 Department of Pathology and Electron Microscopic Center, Harbin Medical University, Harbin 150081, China.; 6 Department of Occupational Health, College of Public Health, Harbin Medical University, Harbin 150081, China.

**Keywords:** chronic cerebral hypoperfusion, mitochondrial complex I deficiency, cognitive impairment, NDUFS8, NRF2

## Abstract

**Rationale:** Chronic cerebral hypoperfusion (CCH), characterized by sustained cerebral ischemic-hypoxic damage due to chronically reduced cerebral blood flow, represents an established risk factor for cognitive decline. While basal forebrain and mitochondrial homeostasis are both vulnerable to hypoperfusion, the precise molecular mechanisms underlying CCH-induced mitochondrial dysfunction in this brain region remain elusive.

**Methods:** Integrating transcriptomic-proteomic analysis of human dementia cohorts to determine the role of NDUFS8 in hypoperfusion-induced cognitive deficits. Stereotaxic injection of AAV vectors encoding *NDUFS8* shRNA or overexpression constructs to induce loss- or-gain-of-function of NDUFS8 in the basal forebrain. Multiscale analyses combining dual-luciferase reporter assays, chromatin immunoprecipitation and computational simulations to reveal the regulatory mechanism of NRF2 on NDUFS8.

**Results:** Our study suggests that NDUFS8 was the most significantly downregulated mitochondrial gene with strong clinical correlation in dementia patients, this was also verified in the basal forebrain of postmortem AD specimens and CCH rats. Basal forebrain-specific NDUFS8 restoration rescued spatial memory deficits in CCH rats through enhancing mitochondrial oxidative phosphorylation function. Mechanistically, multiscale analyses revealed a novel dual regulatory paradigm: NRF2 exhibited impaired binding capacity to both antioxidant response element (ARE) and non-ARE motifs in the *NDUFS8* promoter, while cytoplasmic NRF2 deficiency compromised its stabilizing effect on NDUFS8 protein.

**Conclusions:** Overall, our results indicate that NRF2-NDUFS8 regulatory axis as a major coordinator of mitochondrial homeostasis during hypoperfusion and identify this pathway as a novel therapeutic direction for improving CCH-related cognitive deficits.

## Introduction

With the rapid growth in the human lifespan globally, aging has become an increasingly prominent concern. Cognitive impairment is the most direct manifestation of aging, in which, without early intervention, 15% would eventually progress into dementia after 2 years 1, 2. Among them, Alzheimer's disease (AD) and vascular dementia (VaD) accounts for ~65% and ~20% of dementia respectively that imposes an immense socio-economic burden 3, 4. The phenomenon implies that finding strategies for early intervention is still essential although there is now renewed hope for disease-modifying therapies 5, 6. Chronic cerebral hypoperfusion (CCH), a persistent ischemia-hypoxia status due to chronic shortage of blood supply, which is considered a major driver introducing to early cognitive decline, serving as a pre-clinical phase of AD and VaD patients 7-9. Despite being a state of chronically inadequate brain perfusion 10, CCH induces imbalanced reductions in cerebral blood flow across distinct brain regions in patients both in terms of timing and severity. For example, basal forebrain is the earliest brain area that responds to the reduction of blood supply, with subsequent spread to vulnerable regions including the hippocampus and the cortex 11. Notably, emerging evidence indicates that neurodegeneration in the basal forebrain occurs prior to the cognitive deficits in AD patients 12, making this brain region a focus for understanding the pathogenesis of hypoperfusion-related cognitive impairment.

As the direct target of ischemia-hypoxia, mitochondrial dysfunction presented the hallmark of acute ischemia/reperfusion (I/R) injury, driving neuronal death 13, promoting AD-like pathogenesis, and predisposing patients to marked plaque pathology 14, 15. Mitochondrial oxidative phosphorylation (OXPHOS) entailing five complexes I-V embedded in inner mitochondrial membrane (IMM) is the primary mechanism for cellular energy production and is highly sensitive to changes in mitochondrial function, particularly in neurodegenerative diseases 16. Recent studies demonstrate that reduced mitochondrial complex I activity directly correlated with cognitive deficits 17. NADH dehydrogenase (ubiquinone) Fe-S protein 8 (NDUFS8), a core subunit of complex I, its overexpression effectively rescued rotenone-induced complex I dysfunction in SH-SY5Y cell line 18. Interestingly, the ablation of NDUFS8 drives complex I deficiency induced neurodegeneration in Drosophila model 19, yet the function of NDUFS8 in mammal remains unclear. Despite CCH progression was associated with mitochondrial dysfunction and elevated ROS production in the hippocampus and cortex 20, the changes of NDUFS8 in mitochondria, especially in the basal forebrain following CCH, still remains elusive.

Our findings reveal a selective vulnerability of mitochondrial complex I to CCH in the basal forebrain, with NDUFS8 emerging as the pivotal molecular target. Crucially, targeted restoration of NDUFS8 expression in the basal forebrain effectively rescues CCH-induced cognitive deficits. Furthermore, we unveiled the decline of NRF2, as a transcription factor, not only reduced the transcription of *NDUFS8* gene through the ARE and non-ARE pathway, but also resulted in the destabilize of NDUFS8 to impair its functionality at the protein level. These results indicated that NDUFS8 might serve as a promising biomarker for early diagnosis in CCH-induced cognitive impairment and redefines therapeutic strategies by simultaneously targeting transcriptional regulation and protein stabilization pathways.

## Materials and Methods

### Animals

Forty-Four male Sprague-Dawley rats (2 ~ 3 months old, 280 g ~ 300 g) were obtained from Changsheng Biotechnology (Shenyang, China). All animals for experiments were maintained under standard conditions at 23 ± 1 °C and 55 ± 5% humidity under a 12-hour light and dark cycle. Food and water were available ad libitum. To avoid the influence of estrogen fluctuation in female rats on cognition and mitochondrial function response to hypoperfusion, male rats were used to ensure the consistency of our results. All experimental procedures involving animals were approved by the Institutional Animal Care and Use Committee of Harbin Medical University (Approval Numbers: IACUC-3199620 and IACUC-3052525).

### Human brain and blood samples

Sixteen frozen human post-mortem basal forebrain tissues were acquired from AD patients and age-matched control people who were recruited with informed consent (Table S1). The blood samples of six AD patients were collected from community health examination. The use of human tissue was approved by the Institutional Review Board of the Institute of Basic Medical Sciences of the Chinese Academy of Medical Sciences, China (Approval Numbers: 009-2014 and 031-2017) and the blood samples were approved by Harbin Medical University (Approval Numbers: IRB1008621).

### Permanent bilateral common carotid artery occlusion (2VO)

The 2VO rat model was prepared as described previously 21. Firstly, rats were anesthetized by intraperitoneal injection of 3% pentobarbital sodium (0.15 mL/kg) and maintained with 0.5 ~ 1.0% isoflurane. After anesthesia, bilateral common carotid arteries were permanently ligated using 3-0 silk suture, during which the vagus nerve could not be touched. The common carotid artery was then cut off between the two ligated silk sutures. Sham group underwent the same procedure without ligation. After recovering from anesthesia, rats were returned to their cages. After 8 weeks, brain tissues were collected to perform subsequent experiments.

### Drug administrations

After 2VO surgery, all rats received a one-week recovery. DMF was freshly prepared in 50% PEG-300 solution and administered to 2VO rats by oral gavage daily for 8 weeks. After DMF treatment, 14 randomly selected rats from each group received behavioral test and other experiments. DMF and PEG-300 were purchased from MCE company.

### Stereotaxic injection of the viral vectors

Due to NDUFS8 knockout transgenic mice resulted in complete embryonic lethality prior to organogenesis and the conditional knockout models targeting exons 2-3 of *NDUFS8* only encompass 17.14% of the full coding sequence, potentially compromising functional validity. We chose to establish the loss- or-gain-of-function of NDUFS8 model though stereotaxic injection of AAV vectors carrying either *NDUFS8*-specific shRNA or overexpression constructs into the basal forebrain. The synthesis and AAV packaging of AAV-U6-shRNA (*NDUFS8*), AAV-U6-shRNA (Scramble) and AAV-CMV-*NDUFS8* were performed by BrainVTA before viral injection. Firstly, rats were anesthetized with 3% pentobarbital sodium (0.15 mL/kg) and maintained with 0.5 ~ 1.0% isoflurane according to previous method. Using a 5 μL Hamilton syringe with a 33-gauge tip needle, a volume of 2 μL (10^12^ GC/mL) virus were injected into the basal forebrain region, at a controlled injection speed of 0.5 μL/min. Stereotaxic injection was made at the following coordinates relative to bregma: anterior-posterior (AP), 0.6 mm; medio-lateral (ML), 0.2 mm; dorso-ventral (DV), 6.2 mm below the surface of the dura. These all-used coordinated from the atlases of Paxinos and Watson. After injection, the needle was left in place for 5 mins, and then slowly pulled out to prevent the solution from flowing back. The needle hole was filled with bone wax. Subsequent experiments were performed 8 weeks following the viral injection.

### Morris water maze

Morris water maze (MWM) was performed as previous study. Briefly, it was made up of a black circular pool (2.0 m diameter) that was filled with opaque water by adding black food pigment and maintained at 25 ± 1°C. The pool was divided into four equal quadrants. A hidden platform (20 cm diameter) was submerged 2 cm below the water level. During the cued training (5 days, three trials/day), rats were placed in the water facing the poll wall and given 120 s to find the platform; Rats that failed to find the platform within this time were guided to it and allowed to remain for 20 s. After the final cued trial on day 5, the platform was removed, each rat was subjected to a 120 s probe trial on day 6. Swimming speed (cm/s), escape latency (s), the percentage of swimming time in target quadrant and the number of platform crossings were analyzed in this study. The heat map was generated using MATLAB to visualize the spatial distribution of the mouse's path, with color intensity representing the relative dwell time in different locations. To make the experimental results more realistic, we randomly grouped the experimental animals in the Morris water maze experiment. Similarly, experimental animals that did not move in water or had movement disorders were excluded from this experiment and the rats without cognitive decline were not used for subsequent experiments.

### Laser speckle contrast imaging

Cerebral blood flow (CBF) was monitored non-invasively using laser speckle contrast imaging (LSCI) (Moor Instruments, UK). Rats were anesthetized with 3% pentobarbital sodium (0.15 mL/kg) and maintained with 0.5 ~ 1.0% isoflurane. Using a 0.8-mm diameter drill, the skull was thinned until the blood vessels on the dura are clearly visible. Following surgical preparation, rats were positioned under the LSCI probe. Changes in cerebral blood flow were measured at 8 weeks after 2VO surgery using a perfusion speckle imager (Perimed, Stockholm, Sweden). The CCH rats without the reduction of CBF were not used for subsequent experiments.

### Analysis of RNA-seq data

The human RNA-sequencing (RNA-seq) datasets were obtained from the Aging, Dementia, and Traumatic Brain Injury (TBI) Study in the Allen Brain Institute's human brain disease atlas. This study included 10 control samples and 10 age-matched dementia patients, encompassing AD, VaD and multiple etiologies patients. The proteomics data were retrieved from ProteomeXchange database (PXD027173), comprising 6 AD patients and 6 age-matched control samples 22. Human *miRNAs* expression profile datasets were obtained from GEO database (GSE90828). Differentially expressed genes (DEGs) and proteins were analyzed using the Deseq2 (1.32.0) R package and visualized through volcano plots generated with the ggplot2 package in R v.3.5.2 23. A threshold of |log2FC| ≥ 0.5 and false discovery rate (FDR) adjusted q-value < 0.05 were used to identify significantly differential expressed genes and *miRNAs*.

### Isolation of basal forebrain neurons

The complete brain tissue was quickly put into a sterile ultra-clean bench to ensure neuronal activity. Basal forebrain slices were removed with a blade on the ice according to the location that was shown in the brain atlas and put into the petri dish with pre-cooled phosphate-buffer solution (PBS) in advance. After being cut up with scissors, the slices were transferred into a centrifuge tube containing 0.25% trypsin (6 mL) and transferred into a constant temperature incubator at 37 °C, 120 rpm/min. After 20 mins, the equal amount of culture medium containing serum was added to trypsin to terminate the digestion fully. The supernatant was filtered in 40 μm and centrifuged for 5 mins at 1200 rpm. The cells were precipitated into isolated cells and cultured in neurobasal medium and 2% B27 supplement. The cultured cells were placed in 37 °C with 5% CO_2._

### Primary culture of neonatal rat BFNs

Basal forebrain from postnatal days 1 ~ 3 (P1 ~ 3) rat pups were removed and placed in cold PBS. After dissection and cutting, the tissue was incubated with 0.125% trypsin at 37 °C for 15 mins for digestion. After that, single cells were cultured in DMEM supplemented with 10% fetal bovine serum and 1% penicillin-streptomycin and subsequently inoculated into 6-well plate precoated with 10 μg/mL poly-D-lysine. After incubation for 4 ~ 6 h, the medium was replaced with neurobasal medium containing 2% B27 supplement. The cultured cells were placed in 37 °C with 5% CO_2_. Neurons were collected for experiments following 5 ~ 7 days of culture.

### Cell transfection and drug administration

For *miRNA* transfection,* miR-153* mimics, and NC (scrambled *miR-153*) were synthesized by the Rebio Corporation (Guangzhou, China) and listed in (Table S2). For *siRNA* transfection, si-*NDUFS8* and si-*Nfe2l2* were synthesized by the Rebio Corporation and listed in (Table S3 and Table S4), the negative control for *siRNAs* was provided by the Rebio Corporation (#siN0000001-1-5). These plasmids were transfected into cultured BFNs using X-treme GENE siRNA transfection reagent. For wt-NRF2 and mutant-NRF2 transfection, these plasmids were synthesized by Genescript and transfected into HEK293T cell line (Pricella, Wuhan, China) using Lipo-2000 transfection reagent. The amino acid sequences of wt-NRF2 and mutant-NRF2 were listed in Table S5. DMF, CHX, MG-132 and chloroquine (CQ) were obtained from MCE company and administered them at final concentrations of 10 nM, 50 μM, 5 μM and 10 mM, respectively.

### Western blotting

Total protein lysates were prepared from the basal forebrain of human tissue and rats and primary cultured BFNs with radio immunoprecipitation assay (RIPA) lysis buffer. Nuclear proteins were isolated using a nucleocytoplasmic separation and extraction kit. Total and nuclear protein concentrations were quantified using a BCA Protein Assay Kit with bovine serum albumin as the standard. Protein samples were separated on 10% or 12% sodium dodecyl sulfate-polyacrylamide gel electrophoresis and transferred onto nitrocellulose membranes. Subsequently, the membranes were incubated with primary antibody against NRF2 (#GTX103322, Genetex), NDUFS8 (#GTX114119, Genetex), SDHB (#10620-1-AP, Proteintech), UQCRC2 (#SAB5701523, Sigma), MTCO1 (#1D6E1A8, Invitrogen), ATP5A1 (#14676-1-AP, Proteintech), laminb (#GTX103292, Genetex), β-actin (#AC026, ABclonal) for 4 ℃ overnight. The protein bands were captured using the Odyssey Infrared Imaging System. The signal intensities were analyzed using Odyssey image studio software. The total and nuclear protein were normalized by β-actin and laminb.

### BN-PAGE

100 μg mitochondrial protein isolated from basal forebrain tissue was used to perform BN-PAGE experiment. Briefly, the isolated mitochondria were permeated for 15 mins using an extraction buffer containing 5% digitonin, 4 × BN sample buffer, and protease inhibitors and then centrifuged at 4 ℃, 20,000 g for 40 mins. Next, adding a mixture of glycerol/Coomassie blue G-250 dye (2:1) to the samples to make a sample/mixture ratio of 5:1. Finally, the samples were run on 3% to 12% acrylamide gradient gels at 4 °C.

### Real-time PCR

Total RNA was extracted from the basal forebrain of rats and AD patients, blood of AD patients and BFNs using trizol reagen. By using the ReverTra Ace qPCR RT Kit, the extracted RNA was reversely transcribed into cDNA. qPCR was performed in a volume of 20 μL using FastStart Universal SYBR Green Master. The protocol was as follows: 95 °C for 10 mins; 40 cycles of 95 °C for 15 s, 60 °C for 30 s, 72 °C for 30 s and melt curve analysis. The qRT-PCR primer sequences were designed by Invitrogen and listed in Table S6. Gene expressions were normalized to *U6* and analyzed by δ-δ CT method.

### Mitochondrial morphology assessment

The mitochondrial morphology in primary cultured neurons was stained by 100 nM Mito-Tracker Red CMXRos (#M7512, Invitrogen) for 40 mins in dark. After that, neurons were stored in the culture medium and imaged by a laser confocal microscope (LSM800, Carl Zeiss, Germany). Mitochondrial individuals and branch length were counted by image J software plugin (MINA).

### Immunofluorescence staining

Brain tissues were post-fixed overnight in 4% paraformaldehyde at 4 ℃, dehydrated in 30% sucrose until sinking, and sectioned into 20 μm slices. These slices were permeabilized with 2% Triton X-100 and blocked with 10% goat serum for 1.5 h at room temperature. Subsequently, the slices were incubated overnight at 4 °C with the primary antibody: anti-NeuN mouse mAb (#94403, Cell Signaling Technology), followed by incubation with Alexa Fluor 488-conjugated secondary antibodies (#A-11094, Invitrogen) for 1 h and DAPI staining (#C1005, Beyotime) for 10 mins. Confocal images were captured using a laser confocal microscope (LSM800, Carl Zeiss, Germany) and analyzed using Image-Pro Plus software.

### Chromatin immunoprecipitation assay (ChIP)

Using the UCSC Genome Browser to extract the promotor sequence of *NDUFS8* and then inputted it into the JASPAR database to predict the transcriptional factors. Three binding sites was found by JASPAR between *Nfe2l2* and the promoter of *NDUFS8*. Subsequently, ChIP experiment was performed with primary cultured neurons by using the Imprint ChIP assay kit. The PCR primer sequences are as following: *NDUFS8* ChIP forward [F] primer 1: 5'-AGAGCCTAAGGCAGGCACTTG-3'; *NDUFS8* ChIP reverse[R] primer 1:5'-TATGGATGAGCACTGAGCTTG-3'; *NDUFS8* ChIP forward [F] primer 2:5'-GCAGCCCTTCGCCTCCTGCGGC3'; *NDUFS8* ChIP reverse [R] primer 2:5'-TCAAAAACTGAGGTTCAGAAG-3'; *NDUFS8* ChIP forward [F] primer 3:5'-TCCCACACCACGGGCCTGTTG-3'; *NDUFS8* ChIP reverse [R]primer 3:5'-GATAAGAACAATTTATCACTC-3'. The negative control primer sequences were performed with forward [F] primer: 5'-GGACAAGTCCTTCATGTCCAAG-3'; reverse [R] primer: 5'-CCAAATGTCACATGATTATTG-3'.

### Luciferase reporter assays

Cultured BFNs were co-transfected with reporter plasmid and pcDNA3.1-*NDUFS8* for 48 h to prepare for reporter analyses. Luciferase assays were performed using a Dual-Luciferase Reporter Assay System (#E1910, Promega Corporation). Firefly luciferase activity normalized to Renilla luciferase was considered as an internal control. The luciferase reporter assays are required with the experiment performed in triplicates.

### Co-immunoprecipitation (co-IP) for proteins

Cells were lysed by cell lysis buffer for Western and IP analysis. The protein complex was captured by the Anti-NRF2 antibody (#GTX103322, Genetex) and Anti-rabbit IgG antibody (#7074, Cell Signaling Technology). Protein complex combined with the antibody were precipitated using Protein A/G PLUS-Agarose (#sc-2003, Santa Cruz Biotechnology). Then, the protein sample was subjected to western blot by using Anti-NDUFS8 antibody (#GTX114119, Genetex).

### Transmission electron microscopy

Fresh basal forebrain tissue was taken and cut into slices with a thickness of 500 nm, after following steps of fixation, dehydration, infiltration, embedding, polymerization, sectioning and staining. Finally, the structure of mitochondria in BFNs were observed by electron microscope.

### ATP content assay

The ATP content was measured by using an enhanced ATP assay Kit (#S0027, Beyotime Biotechnology) as described by the experimental specification. Briefly, after treatment of the sample, the relative luciferase activity (RLU) value was measured with luminometer (Promega Corporation, Wisconsin, USA.). RLU was detected at concentrations of 0.01, 0.03, 0.1, 0.3, 1, 3 and 10 µmol/L to prepare a standard curve, which was used to calculate the concentration of ATP.

### Seahorse assay for oxygen consumption rate (OCR) and extracellular acidification rate (ECAR) measurements

Fresh basal forebrain tissues were cut into 100 um slices and seeded in Seahorse XF96 cell culture microplate the day before seahorse assay. Prior to the assay, microplate was washed twice, and media was replaced with XF Assay medium containing 1 mM pyruvate, 2 mM glutamine and 10 mM glucose in 37 ℃ with non-CO_2_ for 1 h. For OCR assay, basal respiration was first to measure, followed by the addition of oligomycin (1 μM), FCCP (2 μM), rotenone/antimycin A (0.5 μM/0.5 μM) to measure ATP production, maximal respiratory, spare respiratory capacity. Respiratory control ratio (RCR) was calculated according to the previous study 24.

For ECAR assay, basal ECAR was first to measure, followed by the addition of glucose solution (10 mM), oligomycin (1.5 μM), and 2-DG (50 mM) to measure determine glycolysis, glycolytic capacity, and glycolytic reserve. Following the assay, protein concentration was quantified using a BCA assay kit for normalization of OCR and ECAR values.

### Measurement of ROS in vitro and in vivo

Cultured BFNs and isolated neurons from rat basal forebrain were stained 30 mins with 20 μmol/L ROS probe (DCFH-DA) (#S0033S, Beyotime Biotechnology) at 37 °C in dark. By using flow cytometry (Beckman Coulter, California, USA), we chose the fluorescence intensity under FITC conditions to reflect the level of ROS in neurons. These data were analyzed by CytExpert 2.2 and Flow Jo 10 software.

### Measurement of mitochondrial membrane potential (MMP)

The MMP of cultured BFNs and isolated neurons from rat basal forebrain were assayed using JC-1 as directed by the manufacturer (#C2003S, Beyotime Biotechnology). Neurons were incubated with JC-1 fluorescent dye (10 μmol/L) under 37 ℃ for 20 mins in dark. The level of MMP was detected by flow cytometry (Beckman Coulter, California, USA) and confocal laser microscopy (LSM800, Carl Zeiss, Oberkochen, Germany).

### Mitochondrial respiratory chain complex I activity assay

Activity of mitochondrial complex I in rat basal forebrain and BFNs were detected by using assay kit (#KTB1850, Abbkine) as directed by the manufacturer. After isolating the mitochondria of tissues and cells, the values of 0 min (A1) and 2 min (A2) at 340 nm wavelength were detected by the microplate tester, and the value of A1 - A2 was the activity of respiratory complex I.

### Computational Modeling

**Structure optimization:** The complete structures of NRF2 and NDUFS8, which are predicted by Alpha Fold 25, and retrieved from the UniprotKB database with uniport ID: Q16236 and O00217, respectively. Implicit solvent molecular dynamics (MD) simulations were then performed to refine the low-confident regions in the structures. The protein was described by the Amber FF19SB force field 26. The hydrogen mass repartitioning was performed to redistribute masses of heavy atoms to their bonded hydrogens 27. This allows a 4 fs time step in the MD simulations. The system was first energy-minimized with 1000 steps of steepest descent algorithm and 9000 steps of conjugate gradients algorithm. The non-bonded interaction cutoff was set as 999 Å, and the SHAKE algorithm was used to restraint the lengths of bonds that have hydrogen involved 28. After minimization, the system was heated from 0 to 300 K using Langevin dynamics with a 3 ps^-1^ collision frequency. The implicit solvent model proposed by Tsui et al was used 29. The MD refinement simulation was 400 ns long, and we show in Figure S6A-B that structures are reaching stable as evidenced by low-fluctuating root mean squared deviations (RMSD) values over the time course. MD simulations were performed by the AMBER20 package.

**Protein-protein docking and molecular dynamics simulations:** The initial binding poses between NRF2 and NDUFS8 were predicted by the ZDOCK webserver 30, using the MD-optimized structures as input. The ZDOCK uses the fast Fourier transform algorithm to search possible binding poses globally, assuming the binding partners are rigid 31. Residues 108-127 and 143-165 from NDUFS8 were specified as contacting residues and the remaining docking parameters were taken as their default values. The top ten poses were subject to 20 ns implicit solvent MD refinement following the mentioned protocol. The MD trajectory was used to calculate the RMSD of backbone atoms, the root mean squared fluctuations (RMSF) of non-hydrogen atoms, and the molecular mechanics Generalized Born surface area (MM-GBSA) binding free energies between NRF2 and NDUFS8. The RMSD was calculated against the first frame in the trajectory, and the RMSF was calculated after aligning the NRF2 protein to that in the first frame. The MMPBSA.py script from Amber 20 was used for MM-GBSA calculations, with the Generalized Bron model proposed by Onufriev et al 32 and an ionic strength of 0.15 M used.

### Statistical analysis

Data are presented as mean ± SEM with *P* < 0.05 considered statistically significant. The *n* numbers in figure legends represent biological replicates or the number of mice and human samples. The two-tailed Student's t test was used to compare two groups with normal distribution criteria. Comparisons across more than two groups were analyzed by one-way ANOVA. Two-way ANOVA in comparison with day × group was used to analyze the escape latency in MWM test. Tukey post hoc tests were used to investigate post hoc analyses with significant main effects. Graphs were generated with GraphPad Prism 9.5 software.

## Results

### NDUFS8 is the key mitochondrial subunit in dementia patients

**Figure 1:**
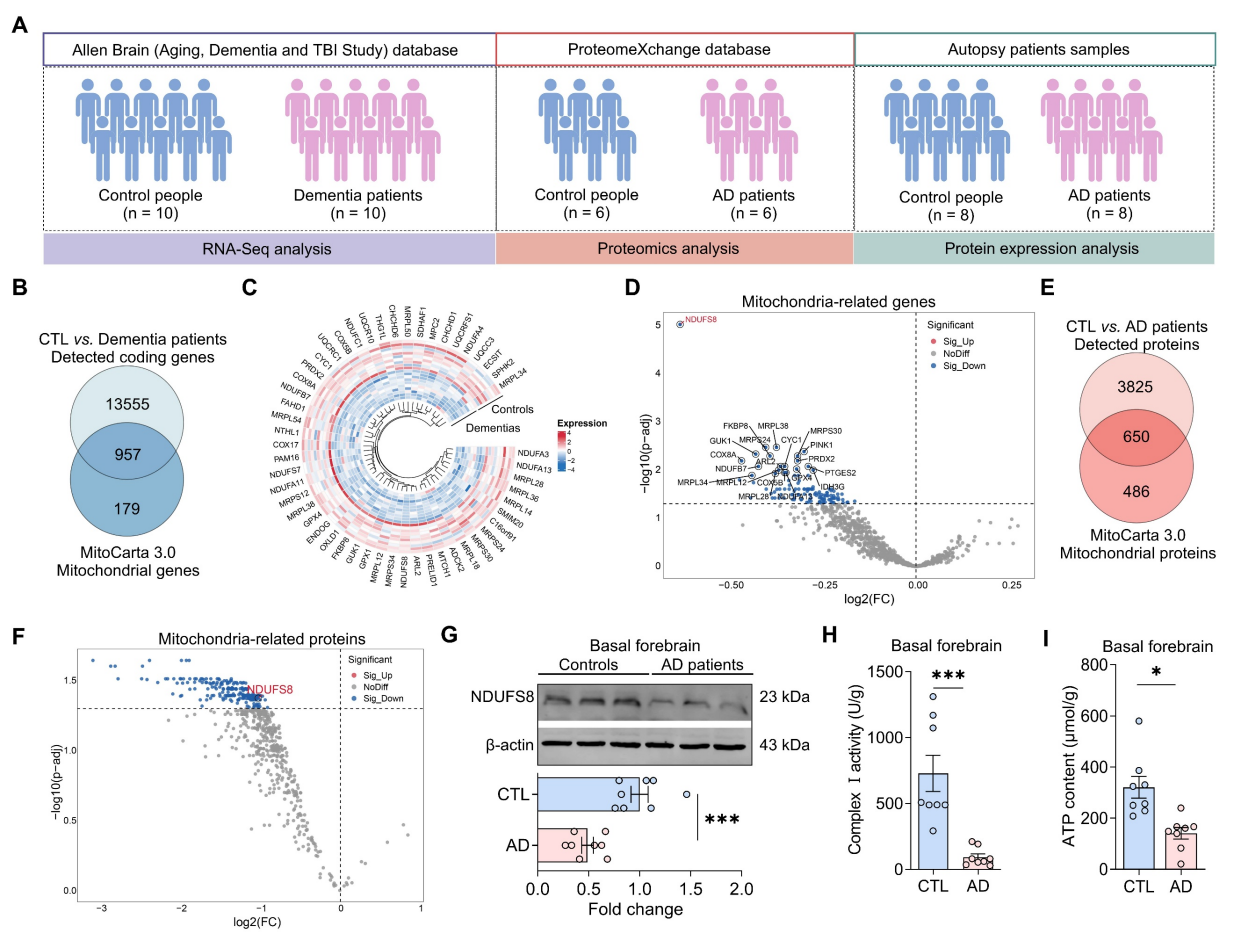
** NDUFS8 is a key factor of AD. (A)** Schematic diagram of patient samples. **(B)** Venn diagram indicated 957 human mitochondrial genes in dementia. **(C)** The heatmap for differentially significant mitochondrial genes. **(D)** Volcano plot showed the differentially expressed mitochondrial genes. **(E)** Venn diagram indicated 616 human mitochondrial proteins in AD patients. **(F)** Volcano plot showed the differentially expressed mitochondrial proteins. **(G)** NDUFS8 expression between control people and AD patients.* n* = 8. Cohen's d = 2.49113. **(H-I)** Decreased mitochondrial complex I activity **(H)** and ATP contents **(I)** in the basal forebrain of AD patients. *n* = 8. Cohen's d = 2.26870 **(H)** and 1.861715087 **(I)**. Data are presented as the mean ± SEM. *P < 0.05, ***P < 0.001.

To investigate the underlying causes of mitochondrial damage in dementia, we screened datasets from Allen Brain human Altas, which provides one of the most authoritative molecular-level maps of the human brain 33. However, identified only a single RNA-sequencing (RNA-seq) dataset available (Figure 1A). Our analysis identified 14512 detected coding genes in the frontal cortex of autopsy samples (Figure 1B, Table S7). When these genes were compared to the 1136 mitochondrial genes from the MitoCarta3.0 database, we identified 957 detected mitochondrial genes (Figure 1B). To determine the most differentially expressed mitochondrial genes (DemtGs) (Figure 1C), NADH dehydrogenase (ubiquinone) Fe-S protein 8 (NDUFS8) exhibited a notably pronounced decrease in dementias compared to controls (Figure 1D), which is a critical indicator of mitochondrial respiratory complex I dysfunction. Next, to further assess the changes of NDUFS8 in patients, we analyzed hippocampal proteomic data collected from ProteinXchange database (Table S8) and surprisingly found a decrease in NDUFS8 expression in the hippocampi of AD patients compared to age-matched controls (Figure 1E-F). This finding was further validated in the basal forebrain of human AD autopsy samples (Figure 1G). Crucially, we also detected a functional decline in both complex I activity and ATP levels in the basal forebrain of post-mortem AD patients compared with the age-matched control subjects (Figure 1H-I). These results implied that loss of NDUFS8 might be a pivotal factor in the process of cognitive decline.

### Dysfunction in mitochondria and NDUFS8 expression in the basal forebrain of CCH rats

**Figure 2:**
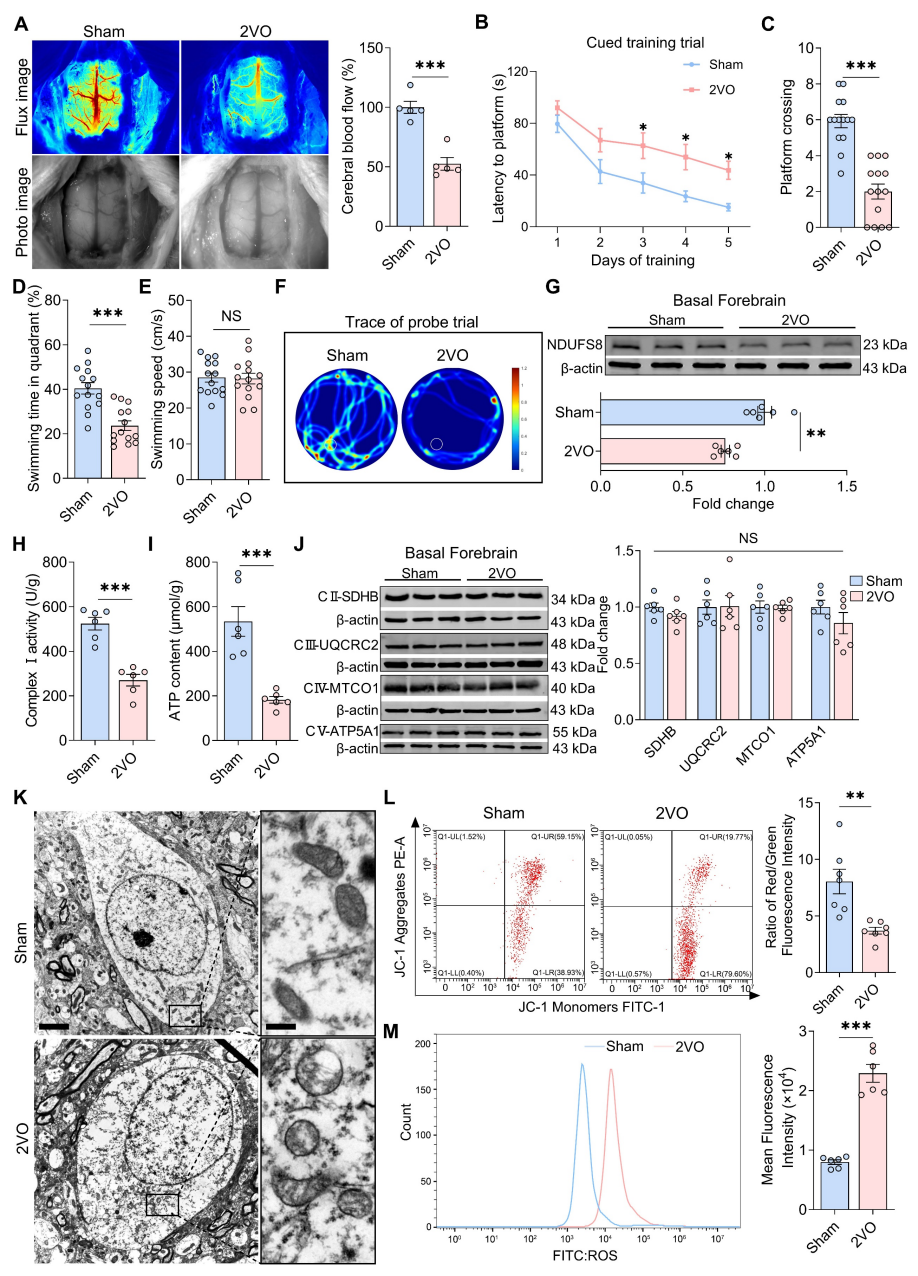
** Impaired mitochondrial function in the basal forebrain of 2VO rats. (A)** Cerebral blood flow was reduced in 2VO rats. *n* = 5. Cohen's d = 4.0704. **(B)** Total escape latency to platform in MWM test. **(C-D)** CCH reduced platform crossings **(C)** and percentage of swimming time **(D)** in the target quadrant. *n* = 14. Cohen's d = 2.65498 **(C)** and 1.88117 **(D)**. **(E)** CCH did not change the swimming speed.* n* = 14.* P* = 0.8831, Cohen's d = 0.05571. **(F)** Representative path tracing on day 6. **(G-I)** CCH reduced NDUFS8 expression **(G)**, mitochondrial complex I activity **(H)** and ATP **(I)** in the basal forebrain. *n* = 6. Cohen's d = 2.80667 **(G)**, 3.78375 **(H)** and 2.98457 **(I)**. **(J)** The protein levels of mitochondrial complex II-V in the basal forebrain. *n* = 6. For SDHB: *P* = 0.8981, Cohen's d = 0.07564; For UQURC2: *P* = 0.1716, Cohen's d = 0.85051; For MTCO1: *P* = 0.9446, Cohen's d = 0.04069; For ATP5A1: *P* = 0.2337, Cohen's d = 0.73163. **(K)** CCH impaired mitochondrial morphology. Scale bar: top: = 2 μm; bottom = 250 nm. *n* = 30 neurons from 6 rats. **(L-M)** CCH decreased JC-1 signal **(L)** and increased intracellular ROS level **(M)** in BFNs. Cohen's d = 2.05391 **(L)** and 5.55312 **(M)**. *n* = 6 ~ 7. Data are presented as the mean ± SEM. **P < 0.01, ***P < 0.001. NS means no significant difference.

To clarify whether NDUFS8 protein expression in basal forebrain is sensitive to hypoperfusion-induced cognitive deficits, we performed 2VO surgery in rats, a CCH rat model as previously reported 34. After 8 weeks, cerebral blood flow was reduced approximately 50% in 2VO rats (Figure 2A). Morris water maze (MWM) test further revealed that CCH significantly increased the time taken by rats to locate the platform in all quadrants during cued learning trials (Figure 2B and Figure S1A-C), and in the probe trail, 2VO rats exhibited fewer platform crossings (Figure 2C) and a reduced percentage of swimming time in target quadrant, independent of swimming speed (Figure 2D-F), indicating memory impairment in 2VO rats. We next verify the mitochondrial function and NDUFS8 expression in the basal forebrain of 2VO rats. There was a significant decline in NDUFS8 level and a substantial decrease in complex I activity in the basal forebrain of these rats with lower ATP level (Figure 2G-I). Acknowledging that, complexes (II-V) are also essential for ATP production 35, we proceeded to evaluate the expression of these complexes using specific marker proteins. As shown in Figure 2J, the levels of these marker proteins for the complex II to V were unchanged between the two groups. Despite preserved expression of complexes II-V, we observed severely vacuolated mitochondria in the basal forebrain neurons (BFNs) of 2VO rats rather than sham rats under transmission electron microscopy (TEM) (Figure 2K), a pathology directly associated with NDUFS8 reduction. Using JC-1 staining to detect early signs of mitochondrial dysfunction 36, we noted a marked decrease in the mitochondrial membrane potential (MMP) in BFNs of 2VO rats (Figure 2L) and supported by a noticeable elevated in reactive oxygen species (ROS) with an approximately 2.5-fold higher (Figure 2M) in the basal forebrain of 2VO rats compared to sham rats. These observations underscore the unique and critical role of NDUFS8 on mitochondrial function in the basal forebrain in CCH.

### Changes of NDUFS8 in BFNs affects spatial memory in rats

**Figure 3:**
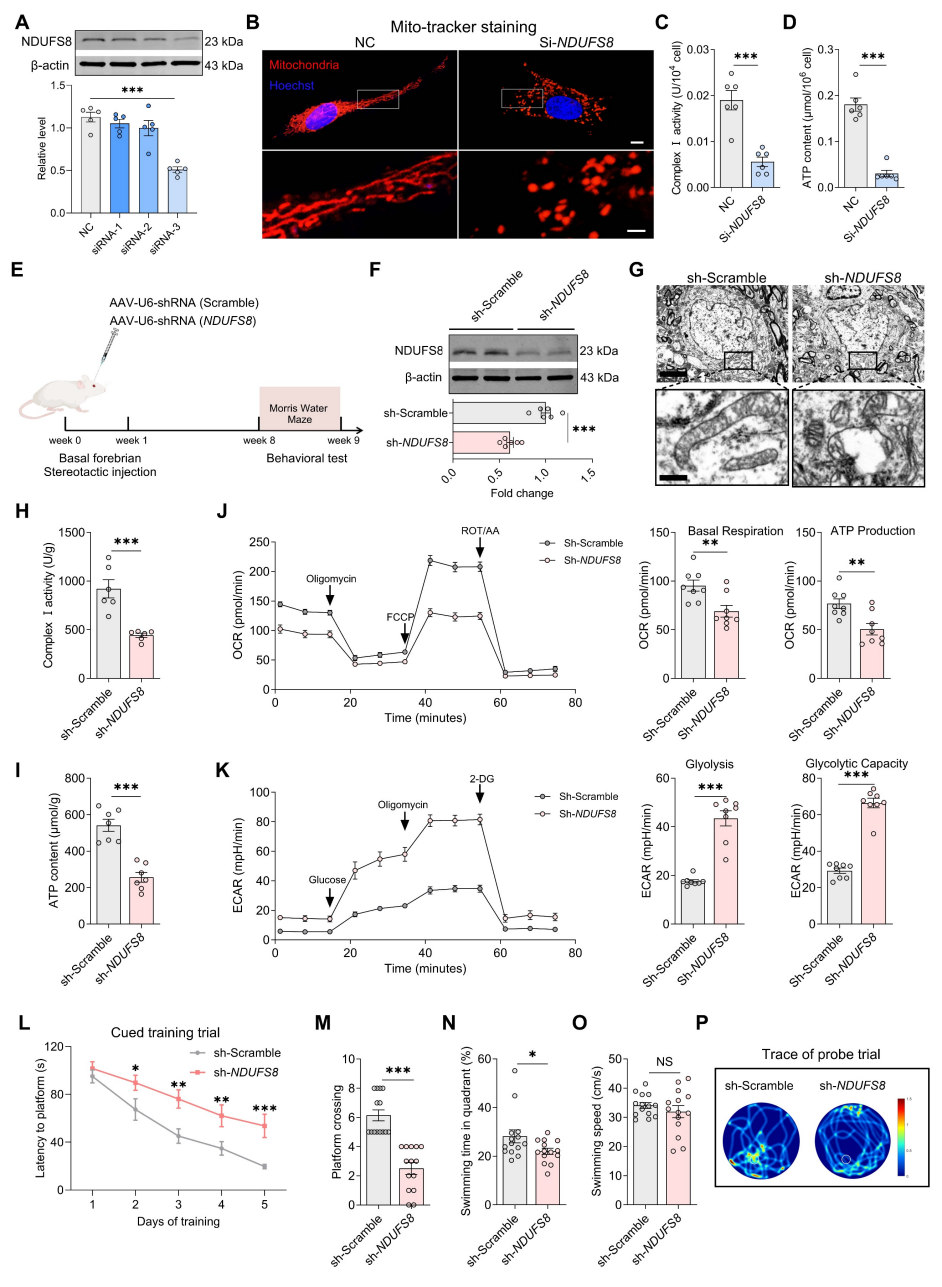
** Loss of NDUFS8 impairs mitochondrial function in BFNs and cognition in rats. (A)** SiRNA-3 transfection reduced NDUFS8 expression in BFNs.* n* = 5. η² = 0.79547. **(B)** The mitochondrial morphology stained with Mito-tracker in BFNs. Scale bar: top: = 10 μm; bottom = 2 μm. **(C-D)** SiRNA-*NDUFS8* transfection impaired mitochondrial complex I activity **(C)** and ATP concentration **(D)** in BFNs. *n* = 6. Cohen's d = 3.30696 **(C)** and 5.44080 **(D). (E)** Schematic diagram of stereotactic injection.** (F)** AAV-sh-*NDUFS8* injected decreased NDUFS8 expression.* n* = 6. Cohen's d = 3.60615. **(G)** Loss of NDUFS8 caused severe mitochondrial vacuolization. Scale bar: top: = 500 nm; bottom = 50 nm. *n* = 30 neurons from 6 rats. **(H-I)** NDUFS8 knockdown reduced mitochondrial complex I activity** (H)** and ATP content **(I)**. *n* = 6 ~ 7. Cohen's d = 2.85358 **(H)** and 3.59783 **(I)**. **(J-K)** NDUFS8 knockdown reduced the OCR levels **(J)** and increased the ECAR levels **(K)**. *n* = 8. Cohen's d = 1.59794 (Basal Respiration), 1.71006 (ATP Production), 4.06605 (Glycolysis) and 6.18066 (Glycolytic Capacity). **(L)** Total escape latency to platform in MWM test. **(M-N)** AAV-sh-*NDUFS8* injection reduced platform crossings **(M)** and percentage of swimming time **(N)** in the target quadrant. *n* = 14. Cohen's d = 2.50057 **(M)** and 2.59444 **(N)**. **(O)** AAV-sh-*NDUFS8* injection did not change the swimming speed.* n* = 14. *P* = 0.3580, Cohen's d = 0.78595. **(P)** Representative path tracing on day 6. Data are presented as the mean ± SEM. *P < 0.05, **P < 0.01, ***P < 0.001. NS means no significant difference.

To establish the causal role of NDUFS8 deficiency in the basal forebrain in cognitive impairment, we first used small interfering RNAs (si) to suppress expression of NDUFS8 in primary cultured BFNs (Figure 3A and Figure S2A). Mito-tracker staining employed to evaluate mitochondrial structure, revealed that NDUFS8 suppression remarkably reduced the length of mitochondria branches and increased the number of mitochondria fragments (Figure 3B and Figure S2B-C), which correlated with functional deficits in diminished complex I activity and ATP level, coupled with an increase in ROS level **(**Figure 3C-D and Figure S2D). We then engineered an adeno-associated virus short hairpin RNA (AAV-*shRNA*) targeting NDUFS8 vector (AAV-sh-*NDUFS8*) and stereotaxically administered it into the basal forebrain of rats over 8 weeks (Figure 3E). Downregulation of NDUFS8 in vivo (Figure 3F) resulted in severe vacuolization of mitochondria (Figure 3G), a reduction in complex I activity and ATP levels (Figure 3H-I), and an elevation in ROS (Figure S3A). To further investigate the specific function of NDUFS8 on mitochondrial metabolism, we used mitochondrial stress test to analysis the oxygen consumption rate (OCR) and extracellular acidification rate (ECAR) as indicators of mitochondrial respiration and glycolytic function, respectively. In comparison to the sh-Scramble group, loss of NDUFS8 in rats resulted in a significant decrease in OCR level, with a notable reduction in basal respiration and ATP production (Figure 3J). Furthermore, ECAR levels also increased in sh-*NDUFS8* rats (Figure 3K), characterized by a marked upregulation in glycolysis and glycolytic capacity. These results indicated that NDUFS8 deficiency in the basal forebrain induced severe disturbances in energy metabolism within mitochondria in rats.

Considering that mitochondrial dysfunction directly damages neurons and spatial memory 37, as predicted, we observed the neuronal loss in the basal forebrain of sh-*NDUFS8* treated rats, evidenced by a reduction in NeuN positive cell counts (Figure S3B), and NDUFS8 knockdown significantly prolonged the escape latency in all quadrants (Figure 3L and Figure S3C-E), decreased platform crossings and the percentage of swimming time in target quadrant, irrespective of swimming speed (Figure 3M-P). These results demonstrate that NDUFS8 deficiency in the basal forebrain suffices to induce cognitive decline through mitochondrial dysfunction. To ensure that the observed mitochondrial function and cognitive deficits were not a result of off-target effects, we predicted the off-target possibility of targeting *NDUFS8* coding sequence “GGACTACACGCTATGACATT” in AAV-*shRNA* using NCBI database (Table S9), which demonstrated the high conservation of the *NDUFS8* gene in this sequence (E value = 1.5). Then we transfected *NDUFS8*-knockdown basal forebrain neurons with an expression plasmid for an shRNA-resistant *NDUFS8* (Res*NDUFS8*) with a codon usage different from the original plasmid. Res*NDUFS8* restored the protein levels of NDUFS8, as well as increase of complex I activity and ATP levels compared to the neurons transfected with sh-*NDUFS8* (Figure S3F-H), further excluding the off-target effects.

**Figure 4:**
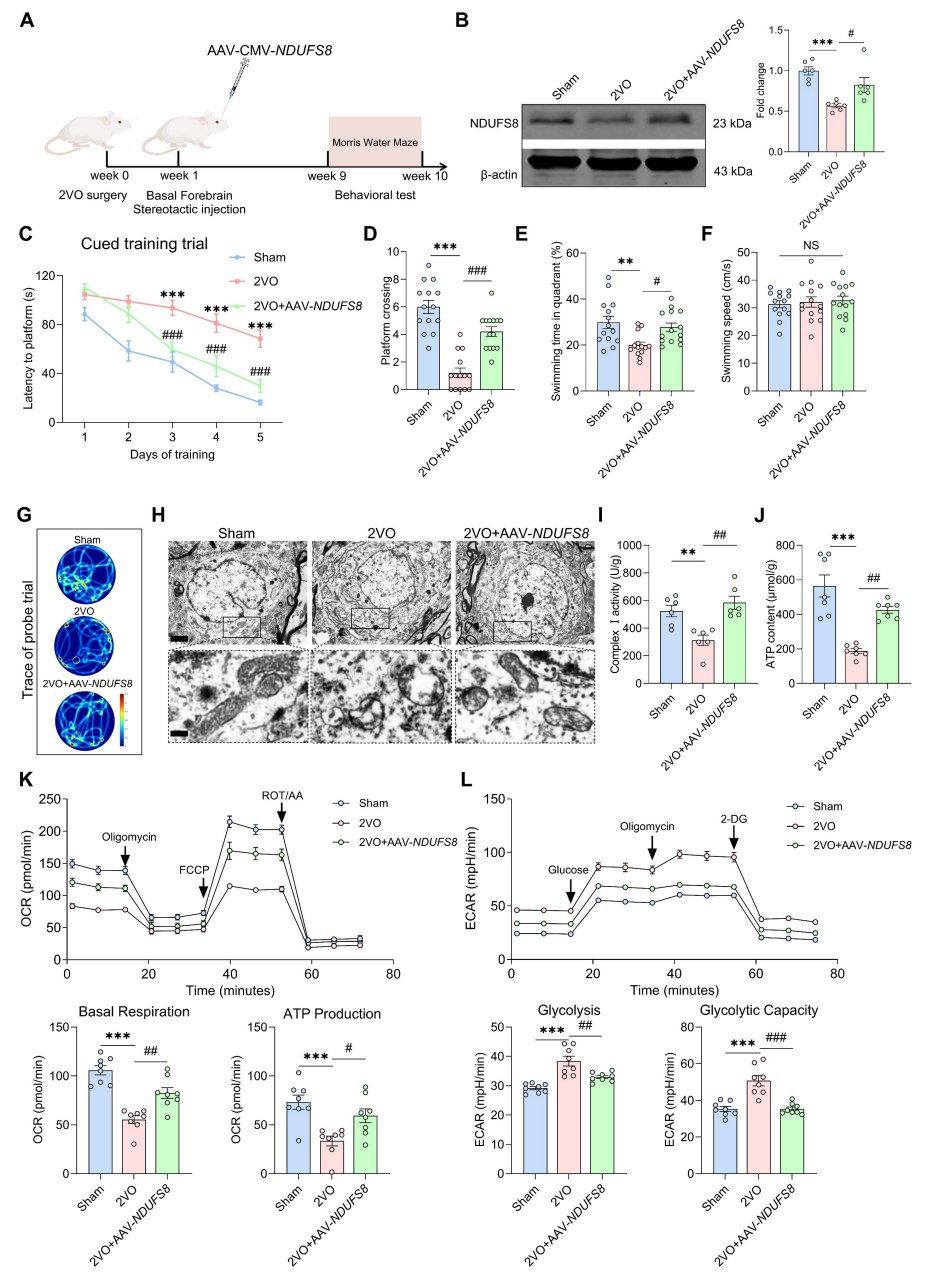
**Recovery of NDUFS8 improves mitochondrial dysfunction and cognitive impairment in CCH rats. (A)** Schematic diagram of stereotactic injection.** (B)** AAV-CMV-*NDUFS8* injection increased NDUFS8 expression in 2VO rats.* n* = 6. η² = 0.62492. **(C)** Total escape latency to platform after NDUFS8 overexpression. *n* = 14. ***P < 0.001 *vs.* Sham rats; ^###^*P* < 0.001 *vs.* 2VO rats. **(D-E)** NDUFS8 overexpression improved platform crossings **(D)** and percentage of swimming time **(E)** in the target quadrant during probe trial in 2VO rats. *n* = 14. η² = 0.65906 **(D)** and 0.25511 **(E)**. **(F)** AAV-CMV-*NDUFS8* injection did not alter the swimming speed. *n* = 14. *P* = 0.8244, η² = 0.00985. **(G)** Representative path tracing on day 6. **(H)** AAV-*NDUFS8* ameliorated mitochondrial vacuolization morphology in 2VO rats. Scale bar: top: = 1 μm; bottom = 150 nm. *n* = 30 neurons from 6 rats. **(I-J)** NDUFS8 overexpression increased mitochondrial complex I activity **(I)** and ATP levels **(J)** in 2VO rats. *n* = 6 ~ 7. η² = 0.60013 **(I)** and 0.72245** (J)**. **(K-L)** Overexpression of NDUFS8 increased the OCR levels **(K)** and decreased the ECAR levels **(L)**.* n* = 8. η² = 0.65387 (Basal Respiration), 0.38122 (ATP Production), 0.64363 (Glycolysis) and 0.67714 (Glycolytic Capacity). Data are presented as the mean ± SEM. **P < 0.01, ***P < 0.001; ^#^*P* < 0.05, ^##^*P* < 0.01, ^###^*P* < 0.001. NS means no significant difference.

We then hypothesized that introducing of exogenous NDUFS8 might potentially reverse the memory deterioration induced by CCH. To test this, we injected AAV-CMV-*NDUFS8* into the basal forebrain of 2VO rats for 8 weeks to induce overexpression of NDUFS8 (Figure 4A-B). NDUFS8 overexpression reversed spatial memory deficits, evidenced by a consistent day-by-day decrease in time to reach platform during cued learning trial (Figure 4C and Figure S4A-C), as well as increased in platform crossings and swimming time in target quadrant during probe trials (Figure 4D-G), regardless of swimming speed (Figure 4F). Accordingly, this intervention not only alleviated mitochondria vacuolization but also significantly enhanced complex I activity and ATP content (Figure 4H-J), decreased the elevated ROS levels and observed an increase in the count of NeuN positive cells in the basal forebrain of 2VO rats (Figure S4D-E). Furthermore, overexpression of NDUFS8 significantly improved the mitochondrial energy metabolism in 2VO rats, manifested as an increase in OCR level (Figure 4K) and a reduction in ECAR level (Figure 4L). To exclude the stoichiometric imbalance caused by overexpression of NDUFS8, we analyzed the assembly of complexes I-V by using blue native polyacrylamide gel electrophoresis (BN-PAGE) and respiratory control ratio, respectively. The level of complex I was increased in the basal forebrain of 2VO rats after NDUFS8 overexpression (Figure S4F). For the resolution of each individual complex I subunit in the second dimensional SDS-PAGE assay, we found that overexpression of NDUFS8 did not affect other subunits (Figure S4G). For respiratory control ratio (RCR), a marker of mitochondrial respiration function, which was significantly reversed in the basal forebrain of 2VO rats subjected to NDUFS8 overexpression (Figure S4H), indicating the improvement in the steady-state level of assembled oxidative phosphorylation. Collectively, all these results strongly suggest that gain-of-function of NDUFS8 exerts a profoundly beneficial effects on cognitive impairment in CCH rats, along with corresponding improvements in mitochondrial function.

### NRF2 regulates NDUFS8 at the transcriptional level in the nucleus

**Figure 5:**
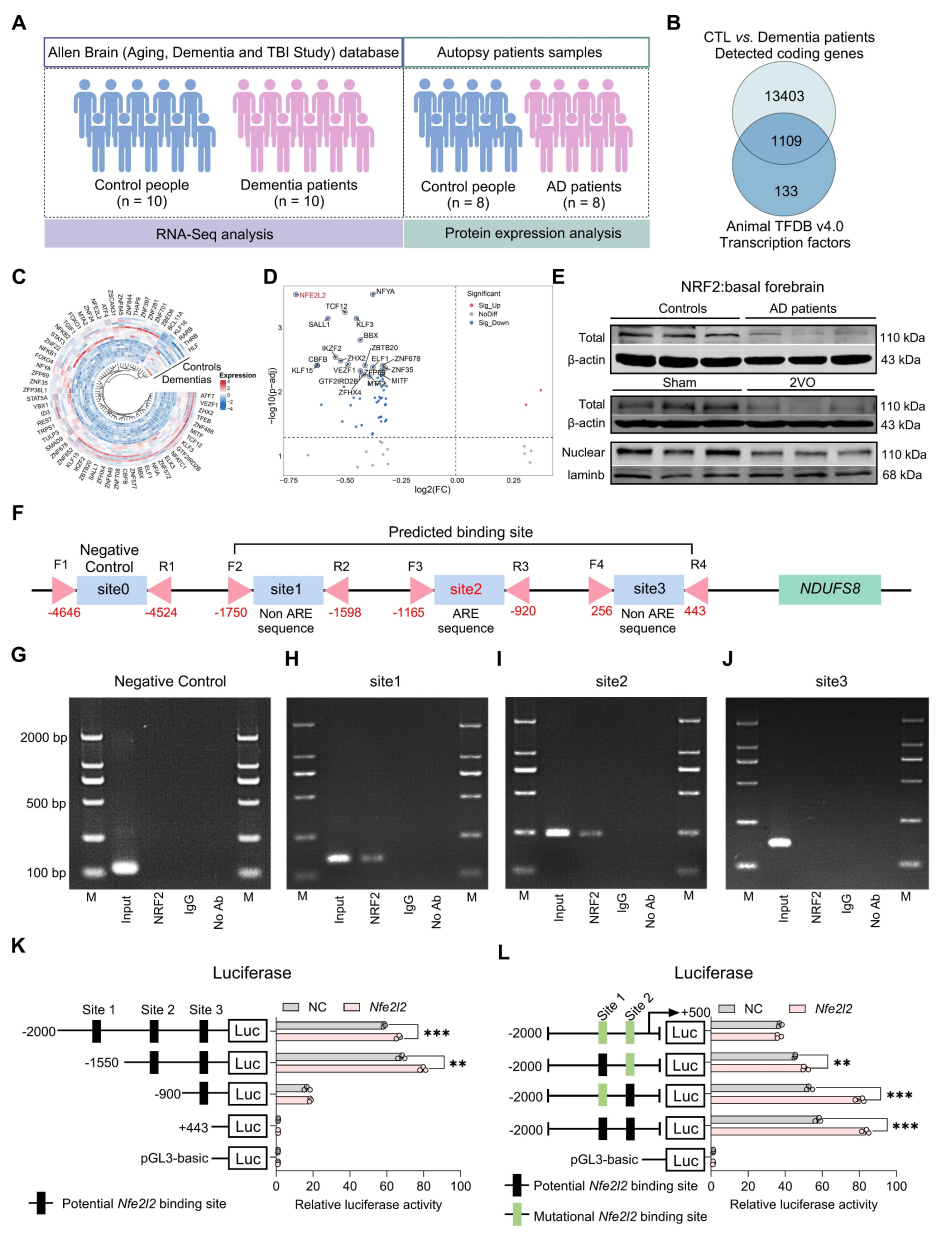
**NRF2 is an upstream transcription factor of *NDUFS8* gene. (A)** Schematic diagram of patient samples. **(B)** Venn diagram indicated 1109 human transcription factors in dementia. **(C)** The heatmap for differentially significant transcription factors. **(D)** Volcano plot representing the most significant differences of genes. **(E)** The total and nuclear protein expression of NRF2 in the basal forebrain of AD patients and 2VO rats.* n* = 8 / 6. **(F)** Schematic representation of the upstream region of the rat *NDUFS8* gene. **(G-J)** ChIP analysis of NRF2 binding to the *NDUFS8* promoter between -4646 bp and 443 bp. **(K-L)** The effect of NRF2 on the three sites **(K)** and mutant site1 and site2 **(L)** of *NDUFS8* promoter activity. *n* = 3. Cohen's d = 6.76533 **(K)** and 14.96458 **(L)**. Data are presented as the mean ± SEM. **P < 0.01, ***P < 0.001*.*

Our next objective was to investigate how CCH leads to the downregulation of NDUFS8 expression. Transcription factors are proteins that orchestrate gene expression in specific cell types 38. In this study, we compared the 14512 coding genes with 1242 transcription factors from the Animal TFDB v4.0 database and identified 1109 detected transcription factors in the frontal cortex of dementia patients (Figure 5A-B). In the differentially expressed transcription factors genes (DetfGs) (Figure 5C), the expression of nuclear factor erythroid 2-related factor 2 (NRF2) showed a most significant decrease compared to other transcription factors (Figure 5D). Given the role of NRF2 in oxidative stress regulation via antioxidant response element (ARE) -dependent pathways 39. We hypothesized NRF2 as a mediator of NDUFS8 suppression under CCH. This was supported by the observation of reduced NRF2 protein levels in the basal forebrain of both AD patients and 2VO rats (Figure 5E and Figure S5A), paralleling NDUFS8 downregulation. Bioinformatics analysis (jaspar.genereg.net) revealed three potential NRF2 binding sites in the promoter region of NDUFS8 (site 1: from -1750 to -1598 bp; site 2: from -1165 to -920 bp; site 3: from 256 to 443 bp) with only site 2 contains classical ARE domain, while the other two are non-ARE domains (Figure 5F and Figure S5B). Surprisingly, using the chromatin immunoprecipitation (ChIP) technique, we found that NRF2 bound to both the ARE domain (site 2) and non-ARE domain (site 1) in the promoter of* NDUFS8* gene (Figure 5G-J and Figure S5C-F). Furthermore, to determine the functional necessity of the identified binding sites, we introduced mutations into the core sequences of site 1 (non-ARE) and site 2 (ARE) in luciferase reporter constructs driven by the *NDUFS8* promoter. Overexpression of *Nfe2l2* activated the wild-type promoter. However, this activation was severely impaired by mutation of either site alone or was completely abolished upon mutation of both sites (Figure 5K-L). Finally, we transfected siRNA-*Nfe2l2* into BFNs and validated the role of NRF2 in the transcriptional activation of *NDUFS8* (Figure S5G-I) that further corroborating our hypothesis.

### NRF2 regulates the stability of NDUFS8 protein within the cytoplasm

**Figure 6:**
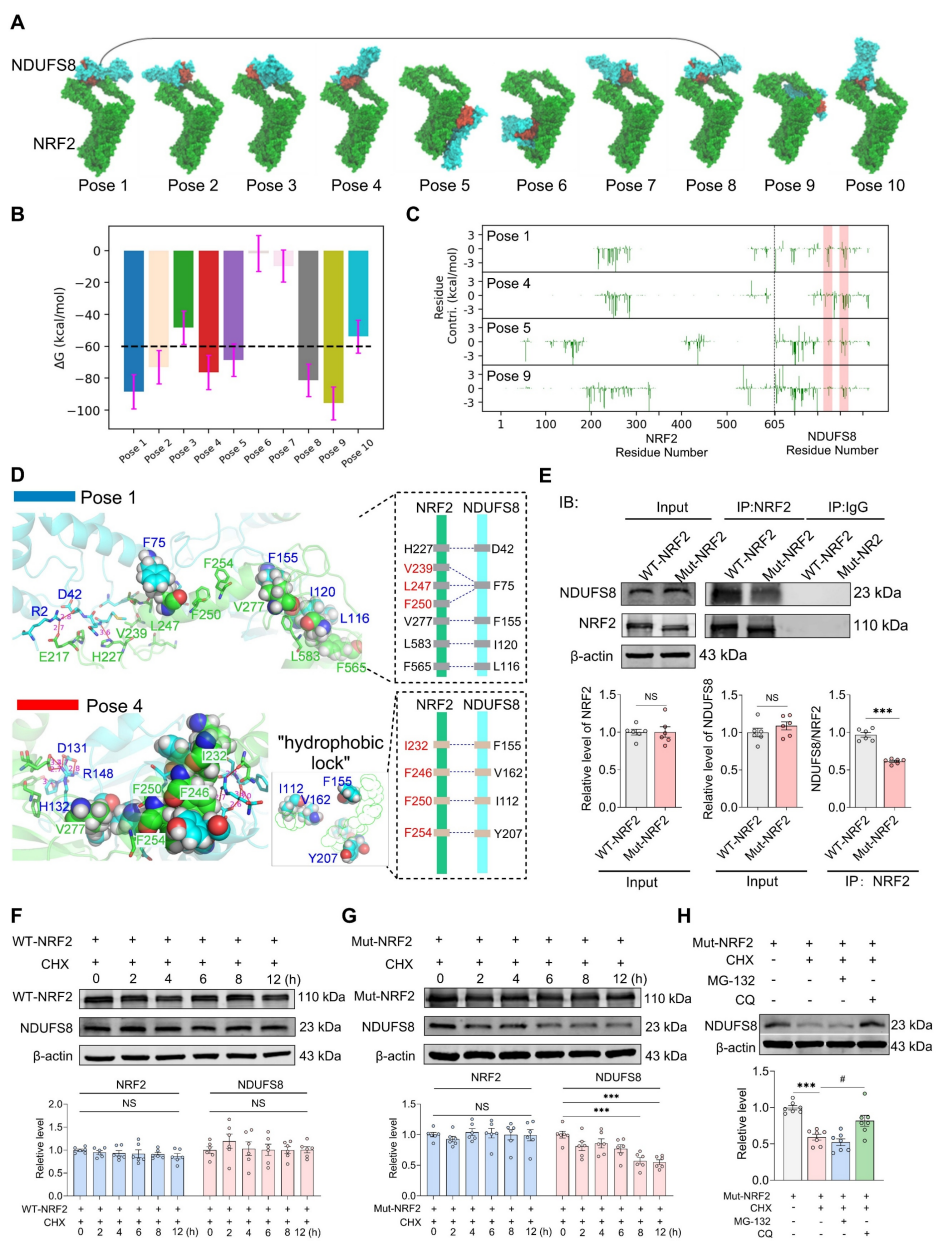
**NRF2 accommodates the binding of NDUFS8 to keep the stability of NDUFS8 protein. (A)** Top ten NRF2-NDUFS8 poses with descending docking scores predicted by ZDOCK. The red area in NDUFS8 depicts the functional regions.** (B)** MMGBSA-calculated binding free energies between NRF2 and NDUFS8 for each pose. **(C)** Per residue energy contribution to MMGBSA-calculated binding free energies. The shaded red regions highlight the two functional regions in NDUFS8. **(D)** Key interactions stabilizing the NRF2-NDUFS8 complex in pose 1 (above) and pose 4 (blow).** (E)** Co-IP analysis between NRF2 and NDUFS8 after transfecting with WT-NRF2 plasmid or Mut-NRF2 plasmid. *n* = 6. For NRF2: *P* = 0.9756, Cohen's d = 0.01464; For NDUFS8: *P* = 0.2862, Cohen's d = 0.65242; For NDUFS8/NRF2: Cohen's d = 5.86055. **(F-G)** The stability of NRF2 or NDUFS8 protein in HEK293T cells transfected with WT-NRF2 plasmid **(F)** and Mut-NRF2 plasmid **(G)** after adding CHX at 0, 2, 4, 6, 8, 12 h.* n* = 6. For NRF2: *P* = 0.6750, η² = 0.09899; For NDUFS8: *P* = 0.8029, η² = 0.07117959 **(F)**; *P* = 0.8974, η² = 0.05054 **(G)**. **(H)** The addition of chloroquine significantly restored the expression of NDUFS8 protein in Mut-NRF2 cells treated with CHX. *n* = 7. η² = 0.69656. Data are presented as the mean ± SEM. ***P < 0.001; ^#^*P* < 0.05*.* NS means no significant difference.

Certain transcription factors have been shown to interact directly with proteins 40; however, whether NRF2 has the same property remained unknown. To this end, we carried out theoretical analyses on the possible interactions between NRF2 and NDUFS8 using computational modelling techniques, targeting two critical functional regions of NDUFS8 (108-127 and 143-165) (Figure S6A-B), which are essential for electrons transfer 41. Since the interaction between NRF2 and NDUFS8 occurs at the amino acid residue level, we employed multi-scale computational simulations. This approach has been demonstrated to provide more precise and high-resolution insights into specific residue-level binding mechanisms, rather than domain-level interactions 42. The results showed that, among the top ten docking poses, poses 1, 2, 3, 4, 7, 8 and 10 exhibited a structurally conserved binding mode characterized by similar NRF2-NDUFS8 interfaces (Figure 6A). Subsequent molecular dynamics (MD) simulations showed that poses 6 and 7 are unstable as evidenced by large structural changes and fluctuations (root mean squared deviations (RMSD) >10 Å and root mean squared fluctuations (RMSF) > 15Å) (Figure S6C-D) and close to zero binding free energies (Figure 6B). Poses 3 and 10 have relatively weak binding (ΔG > -60 kcal/mol) and pose 2, although energetically favorable, is structurally unstable with RMSD > 10 Å and RMSF > 15Å (Figure S6C-D). Among the remaining candidates, pose 8 converged structurally with pose 1 during MD refinement (Figure S6E-F), prompting detailed analysis of poses 1, 4, 5, and 9. In poses 5 and 9, functional residues of NDUFS8 contribute negligibly to binding (Figure 6C), while in poses 1 and 4, these residues are heavily involved in binding by forming polar contacts (hydrogen bonds/salt-bridges) and hydrophobic packings with NRF2 (Figure 6D), especially in pose 4 which residues I112, F155 and V166 from NDUFS8 form a 'hydrophobic lock' to stabilize the hydrophobic chain of NRF2. This notion was indeed supported by carrying out co-IP experiments *in vitro* (Figure S6G) and *in vivo* (Figure S6H), especially in the basal forebrain of 2VO rats, the direct binding of NRF2 and NDUFS8 was significantly reduced compared to sham rats (Figure S6H).

To further elucidate the protein-protein interaction between NRF2 and NDUFS8, we engineered a mutant NRF2 plasmid targeting the 230-270 residues and transfected it into the HEK293T cells. Intriguingly, mutant-NRF2 did not impact either NRF2 or NDUFS8 protein levels (Figure 6E), suggesting that the mutation does not impair NDUFS8 synthesis itself. However, as anticipated, transfection of mutant-NRF2 HEK293T cells significantly impeded NRF2-NDUFS8 interaction compared to the wild-type (WT) NRF2 transfection (Figure 6E). To explored whether NRF2-NDUFS8 binding influences NDUFS8 protein degradation, we employed cycloheximide (CHX), a protein synthesis inhibitor that is used to assess the stability of proteins. Post 8 h of CHX treatment, compared to WT-NRF2, where NDUFS8 levels remained unaltered (Figure 6F), we found a noticeable decline in NDUFS8 levels were observed in HEK293T cells with mutant-NRF2 (Figure 6G). This suggests that diminished NRF2-NDUFS8 interaction adversely affects NDUFS8 protein stability, rather than its synthesis. Notably, NRF2 levels remained consistent across conditions (Figure 6F-G). Furthermore, to delineate the degradation pathway of NDUFS8, we treated mutant-NRF2 cells with CHX for 8 h and co-treated the cells with either the proteasomal inhibitor MG-132 or the lysosomal inhibitor chloroquine (CQ). Co-treatment with CQ almost completely prevented the degradation of NDUFS8 induced by CHX. In contrast, co-treatment with MG-132 failed to rescue the NDUFS8 protein level (Figure 6H), indicating that the instability of NDUFS8 was due to lysosomal degradation.

To summarize, our comprehensive approach, integrating multi-scale computational modeling and co-IP experiments, reveals that NRF2 directly stabilizes NDUFS8 at a specific motif within the Neh7 domain, spanning residues 230-270 (Figure S7). This mode of interaction is crucial for maintaining the stability of NDUFS8 protein.

### Impaired NRF2-NDUFS8 axis is a contributing factor to mitochondrial dysfunction and cognitive decline

**Figure 7:**
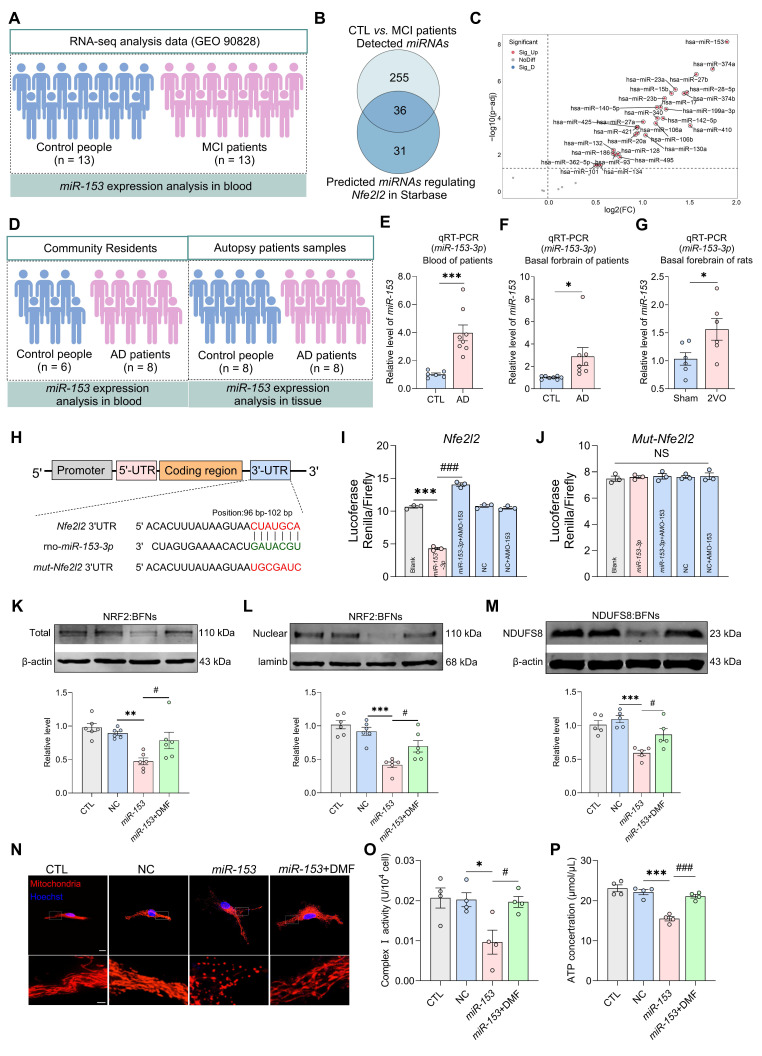
***miR-153* regulates NRF2 and impairs NRF2-NDUFS8 axis in BFNs. (A)** Schematic diagram of patient samples. **(B)** The predicted* miRNAs* which regulate *Nfe2l2* intersected with 291 *miRNAs* from the blood of MCI patients. **(C)** The differentially expressed *miRNAs* were clustered using Volcano plot parameters, the -log10 (FDR p-adj value) and the log_2_ (fold change) are plotted on the y and x axes, respectively. **(D)** Schematic diagram of AD patient samples. **(E-G)** *miR-153* levels in the blood of AD patients **(E)** and in the basal forebrain of AD patients **(F)** and 2VO rats **(G)**. *n* = 6 ~ 8. Cohen's d = 2.57050 **(E)**, 1.18445 **(F)** and 1.35577 **(G)**. **(H)** The sequences of binding and mutation sites of *Nfe2l2* for *miR-153*. **(I)** Transfection of *miR-153* mimics decreased the luciferase activity. *n* = 3. η² = 0.99225.** (J)** Mutation of the binding site eliminated the inhibition of *miR-153* on fluorescence activity. *n* = 3. *P* = 0.9466, η² = 0.06518. **(K-M)** The total **(K)** or nuclear **(L)** protein expression of NRF2 and expression of NDUFS8 **(M)** analysis in BFNs. *n* = 5 ~ 6, η² = 0.56840 **(K)**, 0.72114** (L)** and 0.68450 **(M)**. **(N)** The mitochondrial morphology stained with Mito-tracker in neurons. Scale bar: top: = 10 μm; bottom = 2 μm. *n* = 18 single mitochondria per group from 3 batches of cell culture. **(O-P)** DMF treatment improved mitochondrial complex I activity **(O)** and ATP concentration **(P)** in BFNs. *n* = 4. η² =0.87290 **(O)** and 0.5217 **(P)**. Data are presented as the mean ± SEM. *P < 0.05, **P < 0.01, ***P < 0.001; ^#^*P* < 0.05, ^###^*P* < 0.001. NS means no significant difference.

To ascertain the function of NRF2-NDUFS8 axis in the cognitive deficits observed in CCH rats, we first employed a *microRNA*-based strategy to simultaneously inhibit the entire pathway 43. To identify *miRNA*s most significant associated with hypoperfusion, we analyzed *miRNAs* expression profiles in the blood of patients with mild cognitive impairment (MCI, n = 13) and age-matched healthy controls (n = 13) from the GEO database (Figure 7A, GEO 90828). A total of 291 differentially expressed *miRNAs* were identified between MCI and control samples (Figure 7B). Using the Starbase database, we predicted 67 *miRNAs* that potentially regulate *Nfe2l2* (Figure 7B)**.** Cross-referencing these two datasets revealed 36 *miRNAs* that were both differentially expressed in MCI patients and predicted to target *Nfe2l2* (Figure 7B). From this group, we compiled a list of the top 30 most significantly altered *miRNAs* (Figure 7C). Among which *miR-153* was the most significantly upregulated in MCI patients (Figure 7C).

Based on this finding, we measured *miR-153* levels (Figure 7D) and discovered a significantly increase in the blood of AD patients (Figure 7E), as well as in the basal forebrain of AD patients and 2VO rats (Figure 7F-G) when compared to the age-matched controls. Next, we examined the direct regulatory relationship between *miR-153* and *Nfe2l2* using a dual-luciferase reporter assay. Bioinformatic analysis using the TargetScan Human 8.0, predicted one putative *miR-153-3p* binding site within the 3'UTR region of *Nfe2l2* mRNA, located at position 96-102 bp (Figure 7H). To validate this interaction, we cloned the wild-type *Nfe2l2* 3'UTR into the psiCHECK™-2 vector containing a Renilla luciferase reporter gene and co-transfected it with *miR-153-3p* mimics into primary cultured basal forebrain neurons. Co-transfections of *miR-153-3p* significantly reduced luciferase activity by ~50% compared to the empty vector control, and the effect was reversed by co-transfections AMO-153 (Figure 7I). In contrast, when the binding site was mutated, the inhibitory effect of *miR-153* was completely abolished (Figure 7J). These findings demonstrate that *miR-153* directly targets *Nfe2l2* through a specific binding motif “CUAUGCA” within the 3'UTR.

To verify the influence of NRF2-NDUFS8 axis on mitochondrial function, we transfected *miR-153-3p* mimics into primary cultured BFNs, along with Dimethyl Fumarate (DMF), an agonist of NRF2 with the ability to restore its expression and stabilize the NDUFS8 protein in basal forebrain neurons, resulting in an extension of its half-life from 24 h to approximately 40 h by CHX chase assays (Figure S8A). DMF effectively counteracted the suppression of NRF2 and NDUFS8 expression caused by *miR-153* overexpression (Figure 7K-M). It also increased mitochondria branches length, reduced mitochondria fragments (Figure 7N and Figure S8B-C), upregulated MMP (Figure S8D) and complex I activity (Figure 7O), and decreased ROS level (Figure S8E), ultimately enhanced ATP synthesis (Figure 7P). These findings indicate the disruption of the NRF2-NDUFS8 axis via *miR-153* overexpression in the basal forebrain drives CCH-associated cognitive impairment, primarily by impairing mitochondrial function.

**Figure 8:**
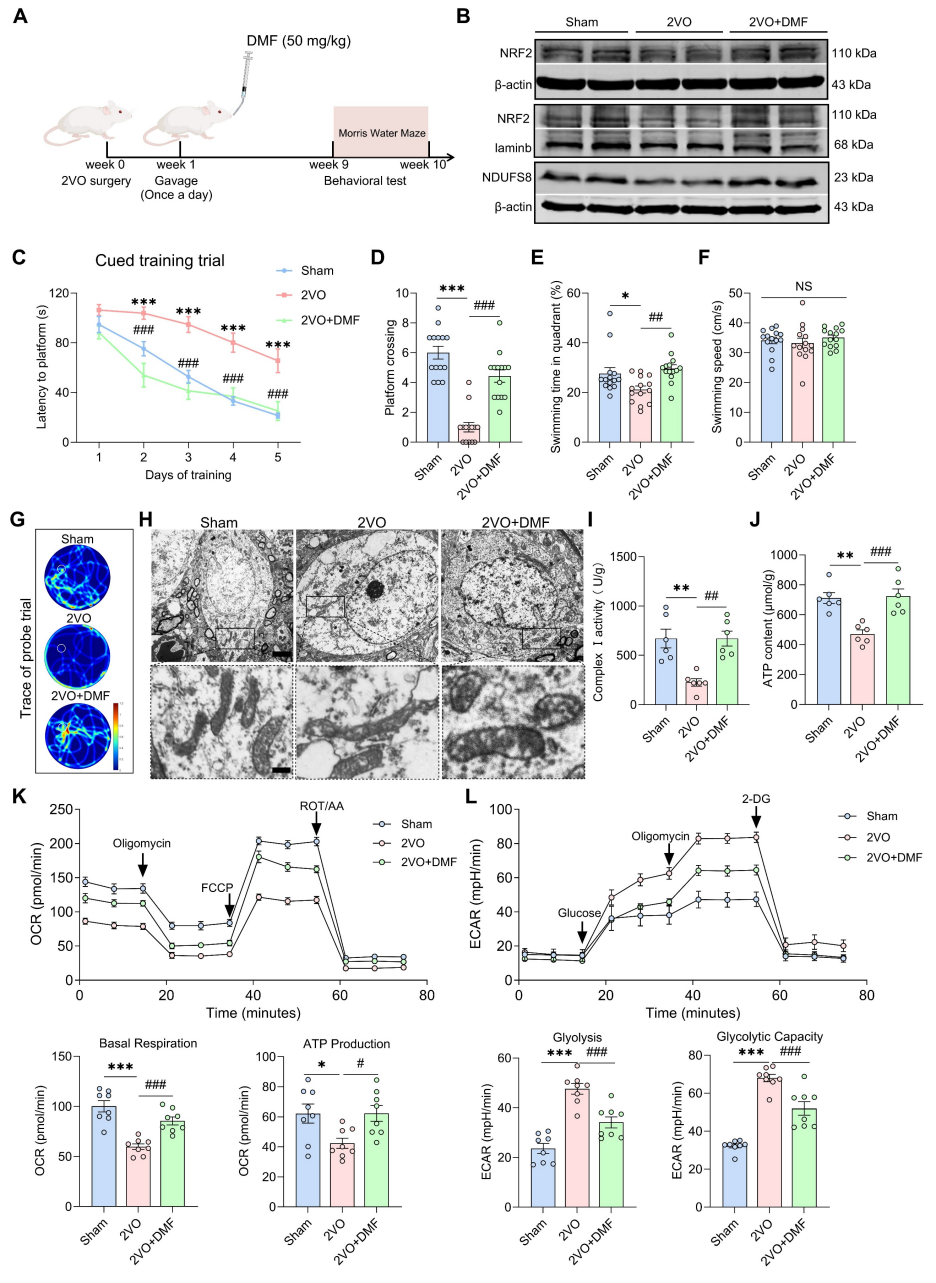
**Recovery of NRF2-NDUFS8 axis improves mitochondrial dysfunction and cognitive impairment in CCH rats. (A)** Schematic diagram of oral administration with DMF.** (B)** DMF treatment increased the total or nuclear protein expression of NRF2 and expression of NDUFS8 in the basal forebrain of CCH rats. **(C-E)** DMF treatment reduced total escape latency to platform **(C)** and increased platform crossings **(D)** and percentage of swimming time **(E)** in the target quadrant during probe trial of 2VO rats.* n* = 14. ***P < 0.001 *vs.* Sham rats; ^###^*P* < 0.001 *vs.* 2VO rats. η² = 0.68692 **(D)** and 0.22806 **(E)**. **(F)** Oral administration with DMF did not alter the swimming speed. *n* = 14. *P* = 0.5704, η² = 0.02838. **(G)** Representative path tracing on day 6. **(H)** DMF treatment ameliorated mitochondrial vacuolization morphology in 2VO rats. Scale bar: top: = 1 μm; bottom = 150 nm. *n* = 30 neurons from 6 rats. **(I-J)** Recovery of NRF2-NDUFS8 axis increased mitochondrial complex I activity **(I)** and ATP levels **(J)** in 2VO rats. *n* = 6. η² = 0.62222 **(I)** and 0.65100** (J)**. **(K-L)** The addition of DMF increased the OCR levels **(K)** and decreased the ECAR levels **(L)**.* n* = 8. η² = 0.76557 (Basal Respiration), 0.43569 (ATP Production), 0.75148 (Glycolysis) and 0.84112 (Glycolytic Capacity). Data are presented as the mean ± SEM. *P < 0.05, **P < 0.01, ***P < 0.001; ^#^*P* < 0.05, ^##^*P* < 0.01, ^###^*P* < 0.001. NS means no significant difference.

We next restored this regulatory axis in 2VO rats by administrating DMF orally for 8 weeks (Figure 8A). DMF treatment enhanced both total and nuclear NRF2 expression as well as NDUFS8 protein levels in the basal forebrain of 2VO rats (Figure 8B and Figure S9A). Behaviorally, DMF treatment remarkably improved the spatial memory performance of 2VO rats, as evidenced by an elevated platform crossings and more time spent in the target quadrant during the probe trial (Figure 8C-G and Figure S9B-D). These improvements occurred without affecting swimming speed (Figure 8F). In addition to rescuing cognitive deficits, DMF alleviated mitochondrial vacuolization (Figure 8H) and restored mitochondrial function, as evidenced by a significant enhancement of complex I activity and ATP levels (Figure 8I-J), reduced ROS accumulation (Figure S9E), and improved bioenergetic balance, reflected by increased OCR levels and decreased ECAR levels (Figure 8K-L). Collectively, our results demonstrate that activation of the NRF2-NDUFS8 axis alleviates cognitive impairment in CCH rats by rescuing mitochondrial dysfunction.

## Discussion

While mitochondrial dysfunction and CCH are recognized preclinical indicators in AD and VaD patients 9, 44, the precise mechanism underlying their association with early cognitive decline remains unclear. Here, we provide the first experimental evidence that CCH induces mitochondrial dysfunction in the basal forebrain through impaired mitochondrial complex I activity, with subsequent cognitive decline being rescued through targeted activation of the NRF2-NDUFS8 axis. For the specific mechanism, our study expands the conventional understanding of NRF2-mediated regulation by revealing two novel mechanisms underlying NDUFS8 protein modulation: a nuclear unreported pathway involving transcriptional regulation independent of ARE, and cytoplasmic post-translational regulation through direct protein-protein interaction. These findings not only establish a dual-target therapeutic manner addressing both nuclear regulation and cytoplasmic protein stabilization but also provide a new direction for developing interventions targeting mitochondrial complex I dysfunction in AD and VaD.

Over the past 40 years, evidence have been accumulating that cerebral bioenergetic failure is a pivotal driver of aging-related neurodegenerative disease, particularly AD and VaD 45, 46. Positioned as central hubs of cellular energy metabolism 16, mitochondrial dysfunction has emerged as a critical driver of Aβ deposition, tau phosphorylation, neuroinflammation, neuronal death and other AD pathology 47, 48. Therefore, regulation of mitochondrial function is a promising direction for future AD and VaD therapy. However, previous studies have generally focused on the mitochondrial function in the hippocampus or cortex, while ignoring the presence of the basal forebrain. The gap in the research work led to the relevant data on the human brain tissue in the basal forebrain are missing from the public database. To ensure the translatability of the research results to the potential of human beings, we screened multi-omics approach integrating RNA sequencing and proteomic profiling of frontal cortex and hippocampus and revealed NDUFS8 deficiency in both the frontal cortex and hippocampi of AD patients, indicating brain-wide alterations as well as consistent changes at both the mRNA and protein levels. Notably, although we did not directly obtain the multi-omics data in the basal forebrain, given that the basal forebrain is mainly supplied with blood by the internal carotid artery 49, the CCH rat model may also preferentially affect the function of basal forebrain. As predicted, the mRNA and protein levels of NDUFS8 in the basal forebrain of 2VO rats were reduced more significantly than those in other regions such as the hippocampus and cortex (Figure S10A-B).

Previous research demonstrates that CCH impaired cholinergic medial septum-diagonal band of Broca-CA1 (MS-dCA1) neurocircuits with declined cognition in rats 50, correlating with loss of ChAT+ and PV+ neurons in the MS 34 However, the mechanism is unknow. In this study, using a comprehensive 'cocktail antibody' approach 51, we discovered that CCH predominantly affects NDUFS8-marked mitochondrial complex I, leading to mitochondrial damage in the basal forebrain without abnormal of mitochondrial complex II - V. Crucially, we found deregulating NDUFS8 elicited impaired spatial memory of normal rats, while upregulating NDUFS8 prevented CCH-induced cognitive decline of 2VO rats. These findings indicate neuroprotective roles of NDUFS8, as evidenced by neuronal degeneration in NDUFS8-deficient Drosophila model 19, and rescue in 2VO rats subjected to NDUFS8 over-expression. Our findings establish NDUFS8-dependent mitochondrial complex I dysfunction may contribute to MS-dCA1circuit failure, potentially linking to cholinergic neuron loss in the basal forebrain of 2VO rats. However, the direct interaction between NDUFS8 and function of MS-dCA1circuit needs to be verified further.

NRF2, a transcriptional factor regulates mitochondrial homeostasis in aging, oxidative stress and age-related diseases by activating cytoprotective genes, all of which contain the ARE sequences in their promoters 39, 52. In the present study, we used the same way of bioinformatics analysis to indicate the specific role of NRF2 in dementia and AD patients. Consistent with the changes of NDUFS8, the effect of CCH on the levels of NRF2 mRNA and protein in the basal forebrain is higher than that in the hippocampus and cortex (Figure S10C-D). Beyond its altered expression, we selected NRF2 as a key transcription factor based on well-established evidence of its protective role against pathological damage and cognitive impairment induced by CCH 53-56, all of which align closely with our findings. Interestingly, a recent study demonstrated that CHK2-mediated phosphorylation of NRF2 at serine 566 and serine 577 enhances its transcriptional activity and antioxidant capacity without affecting its expression 57, whether the post-translational modification function of NRF2 also plays a role in neurodegenerative diseases, remains to be elucidated.

Given the nuclear genomic origin of *NDUFS8* 35, previous studies have focused on antioxidant genes, leaving unresolved whether NRF2 directly regulates mitochondrial-encoded genes. We identified two NRF2 binding sites on the *NDUFS8* gene promoter via ChIP analysis. To investigate the function of these sites, we performed systematic mutational analyses using a dual-luciferase assay rather than constructing a minimal promoter, by generating individual mutations in site1 (-1750 to -1598 bp) and site2 (-1165 to -920 bp), as well as a double mutation in both sites. Specifically, besides the canonical ARE site (-1165-920 bp), there is also a non-ARE motif for NRF2 (-1750-1598 bp), both of which exhibit increased luciferase activity upon NRF2 overexpression, implying a potential non-ARE mechanism for NRF2 regulation of NDUFS8. However, whether NRF2 could regulate other mitochondrial genes via a non-ARE manner requires further exploration. Multi-scale computational modeling predicted a possible specific binding mode between NRF2 and NDUFS8 at the amino acid residue level, forming a “hydrophobic lock” to maintain the stability of NDUFS8 protein. Interestingly, the binding sites of NRF2 are distinct from the previously recognized KEAP1 binding regions (Figure S7) 39, and the effect on the function of NRF2 requires further elucidation. These findings establish that NRF2, traditionally known as a transcription factor, also exerts regulatory control over NDUFS8 at the protein level within the cytoplasm, thus broadening its functional scope beyond mere mRNA level regulation. Tripartite regulatory modes of NRF2 on NDUFS8 in two dimensions appear to explain the reason of why NDUFS8 is more vulnerable in CCH.

In this study, we employed a *microRNA*-based inhibition strategy together with DMF treatment, as described previously 43, to investigate the role of the NRF2-NDUFS8 axis in the CCH rat model. Using bioinformatics analyses and RNA-seq data screening, we identified *miR-153* as the most significantly upregulated *miRNA* targeting *Nfe2l2* in MCI patients. Consistently, *miR-153* levels were increased in the blood of AD patients as well as in the basal forebrain of both AD patients and 2VO rats. Our previous studies have demonstrated that CCH leads to an upregulation of *miR-153* in the hippocampus and cortex of rats, leading to impaired presynaptic neurotransmitter release 58, 59, underscoring the importance of *miR-153* in the context of hypoperfusion-related mechanisms. The regulatory relationship between *miR-153* and *Nfe2l2* was further determined using the dual-luciferase reporter assay. Our findings indicate that inhibiting the NRF2-NDUFS8 axis via *miR-153* overexpression resulted in a substantial alteration in mitochondrial function, which was subsequently restored following administration of DMF. Furthermore, DMF treatment by oral administration in 2VO rats significantly improved the mitochondrial dysfunction in the basal forebrain and cognitive impairment.

This study has a few limitations. In this study, we used adult male SD rats to investigate the detailed molecular mechanisms of mitochondrial dysfunction in response to CCH-induced cognitive impairment. Acknowledged studies have demonstrated that female is easier for live with cognitive decline 60, 61. Whether female also more sensitive to chronic hypoperfusion induced-mitochondrial dysfunction than male will be interest and needs to be clarified.

## Supplementary Material

Supplementary figures and tables.

## Abbreviations

AD: Alzheimer's disease
ARE: antioxidant response element
CCH: chronic cerebral hypoperfusion
CQ: chloroquine
ECAR: extracellular acidification rate
MWM: Morris water maze
NDUFS8: NADH dehydrogenase (ubiquinone) Fe-S protein 8
NRF2: nuclear factor erythroid 2-related factor 2
OCR: oxygen consumption rate
RCR: respiratory control ratio
ROS: reactive oxygen species
VaD: vascular dementia
2VO: bilateral common carotid artery occlusion
