# Supplementary material for: Targeting NDUFS8 in basal forebrain ameliorates cognitive decline related to chronic cerebral hypoperfusion
Source: Theranostics. 2026 Jan 1;16(3):1328–49. doi: 10.7150/thno.117635 (PMC12679367; doi:10.7150/thno.117635)
Supplement: Supplementary file 1 — Supplementary figures and tables. [file thnov16p1328s1.pdf]

Supplementary Material

Targeting NDUFS8 in basal forebrain ameliorates cognitive decline related to chronic cerebral hypoperfusion

Supplementary Tables

Table S1. Clinical details of human samples

Group	Repository ID	Age	Sex	PMI (hours)
CTL	PTB213	70	Male	7.5
CTL	PTB217	72	Male	19.3
CTL	PTB271	70	Male	4.3
CTL	PTB686	72	Male	4.2
CTL	PTB677	70	Male	5.5
CTL	PTB692	72	Male	2.5
CTL	PTB672	74	Male	2.5
CTL	PTB676	62	Male	8.2
AD	PTB344	81	Male	18.0
AD	PTB218	78	Male	13.5
AD	PTB150	84	Female	4.0
AD	PTB702	64	Male	2.8
AD	PTB450	72	Male	23.5
AD	PTB674	72	Male	7.4
AD	PTB436	75	Male	3.0
AD	PTB632	72	Male	6.0

Note: PMI, post-mortem interval

Table S2. Sequences for the Synthesis of *miR-153*.

oligonucleotides	Sequences
miR-153 mimics	sense: 5'-UUGCAUAGUCACAAAAGUGAUC-3'
	antisense: 5'-UCACUUUUGUGACUAUGCAAUU-3'
scrambled *miR-153* (mis-*miR-153*)	sense: 5'-UUCUCCGAACGUGCACGUTT-3'
	antisense: 5'-ACGUGACACGUUCGGAGAATT-3'

Table S3. The sequences of siRNAs for *NDUFS8*.

siRNA	Sequences of primers
siRNA1	sense: 5'-GAAGGCACAGGAACUUGAU-3'
	antisense: 5'-CUUCCGUGUCCUUGAACUA-3'
siRNA2	sense: 5'-ACCCUAAGCUACCUCUUUC-3'
	antisense: 5'-UGGGAUUCGAUGGAGAAAG-3
siRNA3	sense: 5'-GACAUGACCAAGUGUAUCU-3'
	antisense: 5'-CUGUACUGGUUCACAUAGA-3'

Table S4. The sequences of siRNAs for *Nfe2l2*.

siRNA	Sequences of primers
siRNA1	sense: 5'-CAAACAGAAUGGACCUAAA-3'
	antisense: 5'-GUUUGUCUUACCUGGAUUU-3'
siRNA2	sense: 5'-GCAAGAAGCCAGAUACAAA-3'
	antisense: 5'-CGUUCUUCGGUCUAUGUUU-3'
siRNA3	sense: 5'-CGAGAAGUGUUUGACUUUA-3'
	antisense: 5'-GCUCUUCACAAACUGAAAU-3'

Table S5. The amino acid sequences of wt-NRF2 and mutant-NRF2

Proteins		Amino acid sequences	
	1	MMDLELPPPGLPSQQDMDLIDILWRQDIDL	30
	31	GVSREVFDFSQRRKEYELEKQKKLEKERQE	60
	61	QLQKEQEKAFFAQLQLDEETGEFLPIQPAQ	90
	91	HIQSETSGSANYSQVAHIPKSDALYFDDCM	120
	121	QLLAQTFPFVDDNEVSSATFQSLVPDIPGH	150
	151	IESPVFIATNQAQSPETSVAQVAPVDLDGM	180
	181	QQDIEQVWEELLSIPELQCLNIENDKLVET	210
WT-NRF2	211	TMVPSPEAKLTEVDNYHFYSSIPSMEKEVG	240
	241	NCSPHFLNAFEDSFSSILSTEDPNQLTVNS	270
	271	LNSDATVNTDFGDEFYSAFIAEPSISNSMP	300
	301	SPATLSHSLSELLNGPIDVSDLSLCKAFNQ	330
	331	NHPESTAEFNDSDSGISLNTSPSVASPEHS	360
	361	VESSSYGDTLLGLSDSEVEELDSAPGSVKQ	390
	391	NGPKTPVHSSGDMVQPLSPSQGQSTHVHDA	420
	421	QCENTPEKELPVSPGHRKTPFTKDKHSSRL	450
	451	EAHLTRDELRAKALHIPFPVEKIINLPVVD	480
	481	FNEMMSKEQFNEAQLALIRDIRRRGKNKVA	510
	511	AQNCRKRKLENIVELEQDLDHLKDEKEKLL	540
	541	KEKGENDKSLHLLKKQLSTLYLEVFSMLRD	570
	571	EDGKPYSPSEYSLQQTRDGNVFLVPKSKKP	600
	601	DVKKN	630
	1	MMDLELPPPGLPSQQDMDLIDILWRQDIDL	30
	31	GVSREVFDFSQRRKEYELEKQKKLEKERQE	60
	61	QLQKEQEKAFFAQLQLDEETGEFLPIQPAQ	90
	91	HIQSETSGSANYSQVAHIPKSDALYFDDCM	120
	121	QLLAQTFPFVDDNEVSSATFQSLVPDIPGH	150
	151	IESPVFIATNQAQSPETSVAQVAPVDLDGM	180
	181	QQDIEQVWEELLSIPELQCLNIENDKLVET	210
Mutant-NRF2	211	TMVPSPEAKLTEVDNYHFYSSAPSMEKEAG	240
	241	NCSPHAANAAEDSASSILSTEDPNQLTAAA	270
	271	LNSDATVNTDFGDEFYSAFIAEPSISNSMP	300
	301	SPATLSHSLSELLNGPIDVSDLSLCKAFNQ	330
	331	NHPESTAEFNDSDSGISLNTSPSVASPEHS	360
	361	VESSSYGDTLLGLSDSEVEELDSAPGSVKQ	390
	391	NGPKTPVHSSGDMVQPLSPSQGQSTHVHDA	420
	421	QCENTPEKELPVSPGHRKTPFTKDKHSSRL	450
	451	EAHLTRDELRAKALHIPFPVEKIINLPVVD	480
	481	FNEMMSKEQFNEAQLALIRDIRRRGKNKVA	510
	511	AQNCRKRKLENIVELEQDLDHLKDEKEKLL	540
	541	KEKGENDKSLHLLKKQLSTLYLEVFSMLRD	570
	571	EDGKPYSPSEYSLQQTRDGNVFLVPKSKKP	600
	601	DVKKN	630

Note: Mutant amino acids are marked in red

Table S6. Sequences of human and rat primers used for real-time PCR.

miRNAs	Sequences of primers
Hsa-*miR-153-3p*	RT:5’-GTCGTATCCAGTGCAGGGTCCGAGGTATTC GCACTGGATACGACGATCAC-3’<br>Forward: 5’-CGCCGCTTGCATAGTCACAAAA-3’<br>Reverse: 5’-ATCCAGTGCAGGGTCCGAGG-3’
Hsa-*U6*	RT:5’-CGCTTCACGAATTTGCGTGTCAT-3’<br>F: 5’-GCTTCGGCAGCACATATACTAAAAT-3’<br>R: 5’-CGCTTCACGAATTTGCGTGTCAT-3’
Rno-*miR-153-3p*	RT:5’- GTCGTATCCAGTGCGTGTCGTGGAGTCGGC AATTGCACTGGATACGACGATCAC-3’<br>F: 5’-CCGGTTGCATAGTCACAAAAGTG-3’<br>R: 5’-ATCCAGTGCAGGGTCCGAGG-3’
Rno-*U6*	RT:5’-CGCTTCACGAATTTGCGTGTCAT-3’<br>F:5’-GCTTCGGCA-GCACATATACTAAAAT-3’<br>R:5’-CGCTTCACGAATTTGCGTGTCAT-3’
Rno-*NDUFS8*	F: 5’-GTTGACGCTATCGTGGAGGG-3’<br>R: 5’-GGTAGTCAGCCTGGATGTTGG-3’
Rno-*Nfe2l2*	F: 5’-AAACATTCAAGCCGATTAG-3’<br>R: 5’-ATTGCTCCTTGGACATCA-3’
Rno-*ACTB*	F: 5’-CCTGTGGCATCCATGAAACTAC-3’<br>R: 5’-CCAGGGCAGTAATCTCCTTCTG-3’

Table S7. Clinical details of age-matched control people and dementia patients in RNA-seq data

Group	Age	Sex	Clinical diagnosis
CTL	95-99	Female	No Dementia
CTL	90-94	Female	No Dementia
CTL	78	Male	No Dementia
CTL	85	Male	No Dementia
CTL	90-94	Female	No Dementia
CTL	89	Male	No Dementia
CTL	89	Female	No Dementia
CTL	81	Male	No Dementia
CTL	86	Male	No Dementia
CTL	95-99	Female	No Dementia
Dementia	90-94	Male	Alzheimer’s disease
Dementia	77	Male	Others
Dementia	100+	Female	Alzheimer’s disease
Dementia	90-94	Male	Multiple Etiologies

Dementia	89	Female	Vascular
Dementia	89	Female	Alzheimer’s disease
Dementia	100+	Female	Alzheimer’s disease
Dementia	85	Male	Alzheimer’s disease
Dementia	88	Male	Alzheimer’s disease
Dementia	87	Female	Multiple Etiologies

Note: Data derived from Allen Brain Altas

Table S8. Clinical details of age-matched control people and AD patients in proteomics data.

Group	Age	Sex	PMI (hours)
CTL	85	Female	8.0
CTL	75	Male	5.0
CTL	64	Male	4.0
CTL	54	Male	2.0
CTL	72	Female	2.0
CTL	79	Male	2.0
AD	80	Male	5.5
AD	78	Male	13.5
AD	94	Female	4.0
AD	79	Male	6.0
AD	91	Female	8.0
AD	91	Female	4.0

Note: Data derived from ProteomeXchange database

Table S9. Prediction of off-target effect of ShRNA-*NDUFS8* by NCBI database

Description	Scientific Name	Total Score	Query Cover	E value	Acc. Len	Accession
NADH:ubiquinone oxidoreductase core subunit S8 (Ndufs8), mRNA; nuclear gene for mitochondrial product	Rattus norvegicus	40.1	100%	1.5	773	NM_0011063 22.2

Supplementary Figures and Figure legends

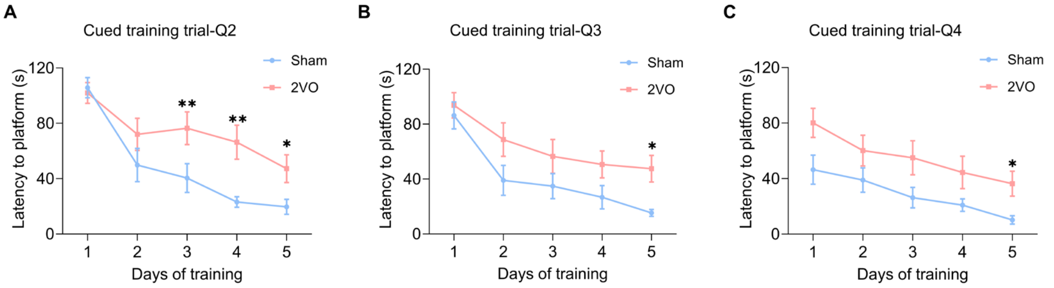

Figure S1. CCH impairs cognitive function in rats. (A-C) CCH increased mean daily latency to locate the hidden platform (Q2/Q3/Q4). *n* = 14. Data are presented as the mean ± SEM. **P* < 0.05, ***P* < 0.01.

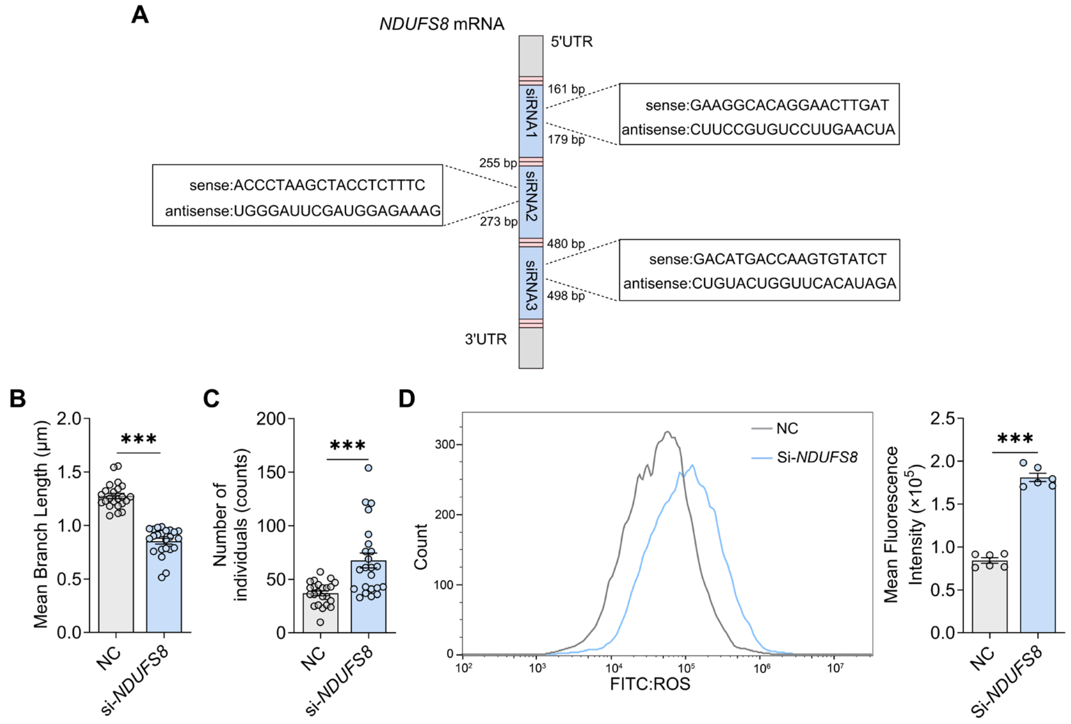

Figure S2. NDUFS8 knockdown impairs mitochondrial morphology and function in BFNs. (A) Schematic diagram of siRNA-*NDUFS8* design. **(B-C)** SiRNA-*NDUFS8* transfection decreased mean branch length of mitochondria **(B)** and increased number of mitochondrial individuals **(C)** in BFNs. n = 23 single mitochondria per group from 3 batches of cell culture. Cohen's d = 3.40479 **(B)** and 1.23271 **(C)**. **(D)** SiRNA-*NDUFS8* transfection increased intracellular ROS level in BFNs. n = 6. Cohen's d = 9.74974. Data are presented as the mean ± SEM. ***$P < 0.001$.

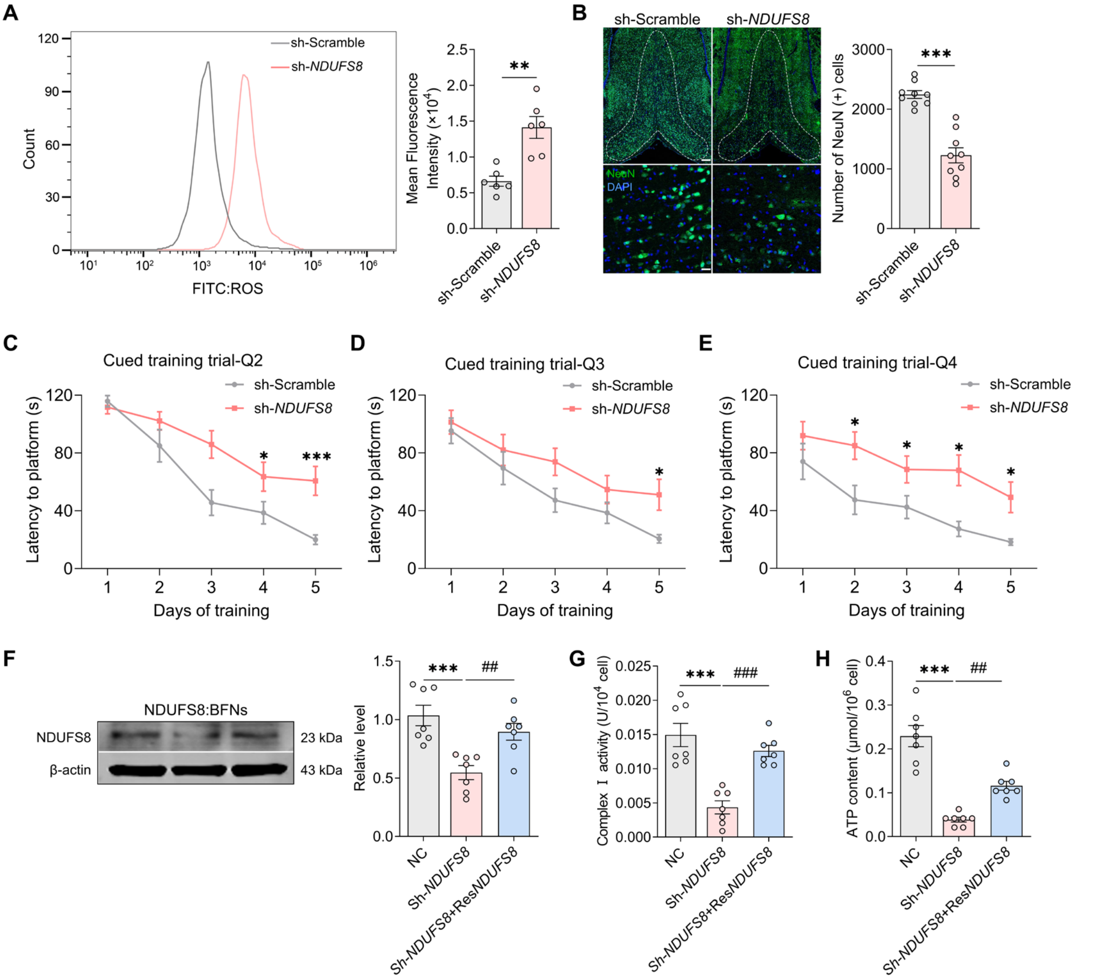

Figure S3. AAV-*NDUFS8* impairs mitochondrial function and cognition in rats. (A) NDUFS8 knockdown in the basal forebrain increased intracellular ROS in rats. n = 6. Cohen's d = 2.59444. **(B)** Decreased counts of NeuN (+) cells in the basal forebrain of AAV-sh-*NDUFS8* rats. Scale bar: above: = 200 μm; below = 20 μm. n = 9 slices from 3 rats. Cohen's d = 3.36708. **(C-E)** AAV-sh-*NDUFS8* injection increased mean daily latency to locate the hidden platform (Q2/Q3/Q4). n = 14. **(F-H)** shRNA-resistant *NDUFS8* (Res*NDUFS8*) increased the expression of NDUFS8 **(F)**, complex I activity **(G)** and ATP levels **(H)** in BFNs transfected into shRNA-*NDUFS8*. n = 7. η^2 = 0.56262 **(F)**, 0.69685 **(G)** and 0.81191 **(H)**. Data are presented as the mean ± SEM. $^{*}P < 0.05$, $^{**}P < 0.01$, $^{***}P < 0.001$; $^{\#\#}P < 0.01$, $^{\#\#\#}P < 0.001$.

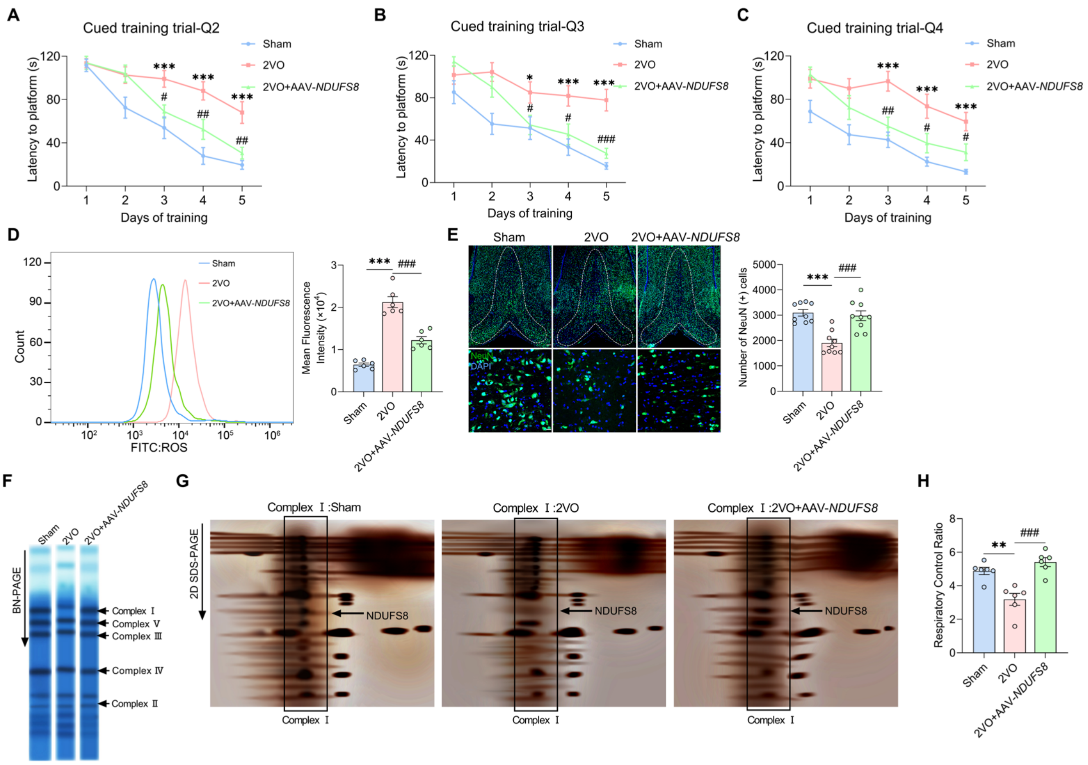

Figure S4. NDUFS8 overexpression improves mitochondrial dysfunction and cognitive decline in 2VO rats. (A-C) AAV-*NDUFS8* injection in the basal forebrain of 2VO rats decreased mean daily latency to locate the hidden platform (Q2/Q3/Q4). *n* = 14. $^{*}P < 0.05$, $^{***}P < 0.001$ *vs.* Sham rats; $^{\#}P < 0.05$, $^{\#\#}P < 0.01$, $^{\#\#\#}P < 0.001$ *vs.* 2VO rats. **(D)** NDUFS8 over-expression decreased intracellular ROS level in 2VO rats. *n* = 6. $\eta^2 = 0.89506$. **(E)** AAV-CMV-*NDUFS8* treated increased counts of NeuN (+) cells in the basal forebrain of 2VO rats. Scale bar: above: = 200 μm; below = 20 μm. *n* = 9 slices from 3 rats. $\eta^2 = 0.58632$. **(F)** Coomassie Brilliant Blue staining of BN-PAGE with indicated positions and quantifications of mitochondrial complexes I to V. *n* = 3. **(G)** Two-dimensional Blue Native/SDS–PAGE of complexes I. *n* = 3. **(H)** NDUFS8 over-expression increased respiratory control rate in 2VO rats. *n* = 3. $\eta^2 = 0.68910$. Data are presented as the mean ± SEM. $^{**}P < 0.01$, $^{***}P < 0.001$; $^{\#\#\#}P < 0.001$.

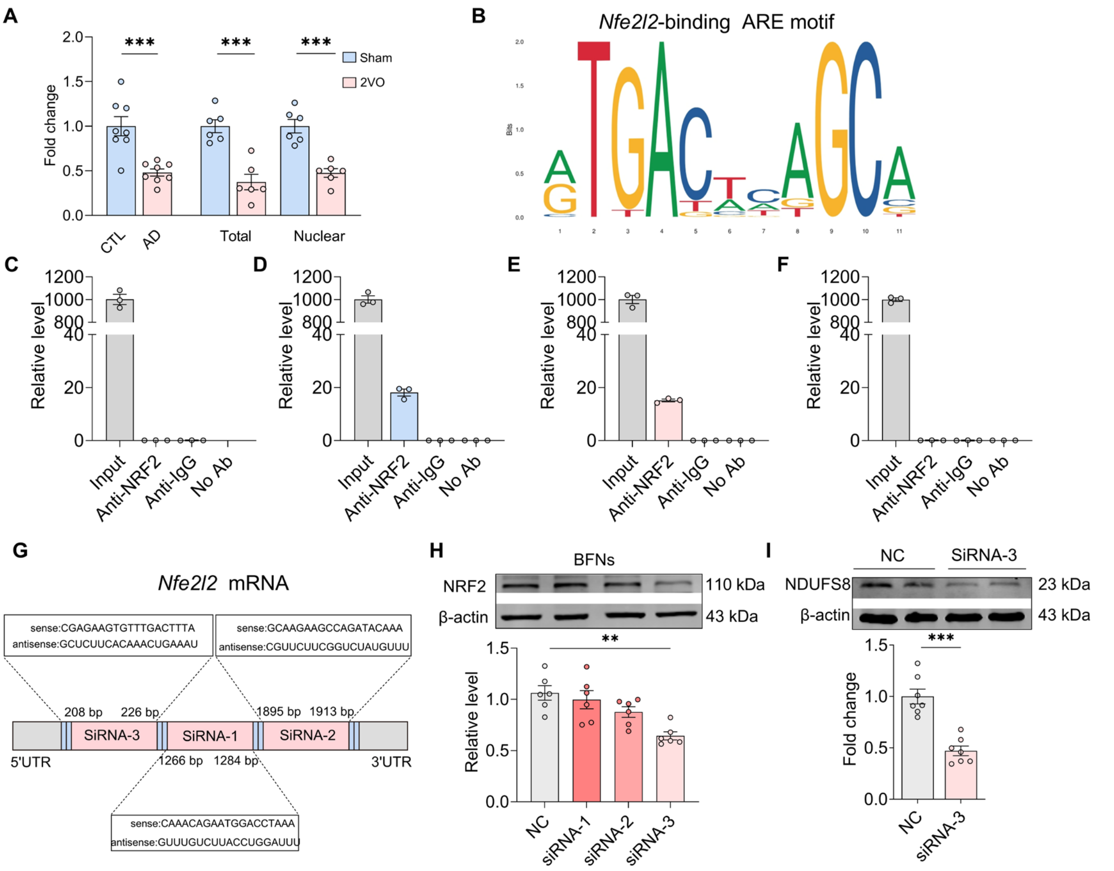

Figure S5. NRF2 is an upstream transcription factor of *NDUFS8* gene and regulates its expression. (A) Decreased protein of NRF2 in the basal forebrain of AD patients and 2VO rats. $n = 6 \sim 8$. For CTL&AD: Cohen's d = 2.27287; For total protein: Cohen's d = 3.18819; For nuclear protein: Cohen's d = 3.33041. **(B)** Statistical diagram of *Nfe2l2*-binding ARE motif. **(C-F)** qPCR analysis of NDUFS8 binding sequences. $n = 3$. **(G)** Schematic diagram of SiRNA-*Nfe2l2* design. **(H)** SiRNA-3 transfection decreased the expression of NRF2 in BFNs. $n = 6$. $\eta^2 = 0.54199$. **(I)** Loss of NRF2 decreased NDUFS8 expression in BFNs. $n = 7$. Cohen's d = 3.28934. Data are presented as the mean ± SEM. $^{**}P < 0.01$, $^{***}P < 0.001$.

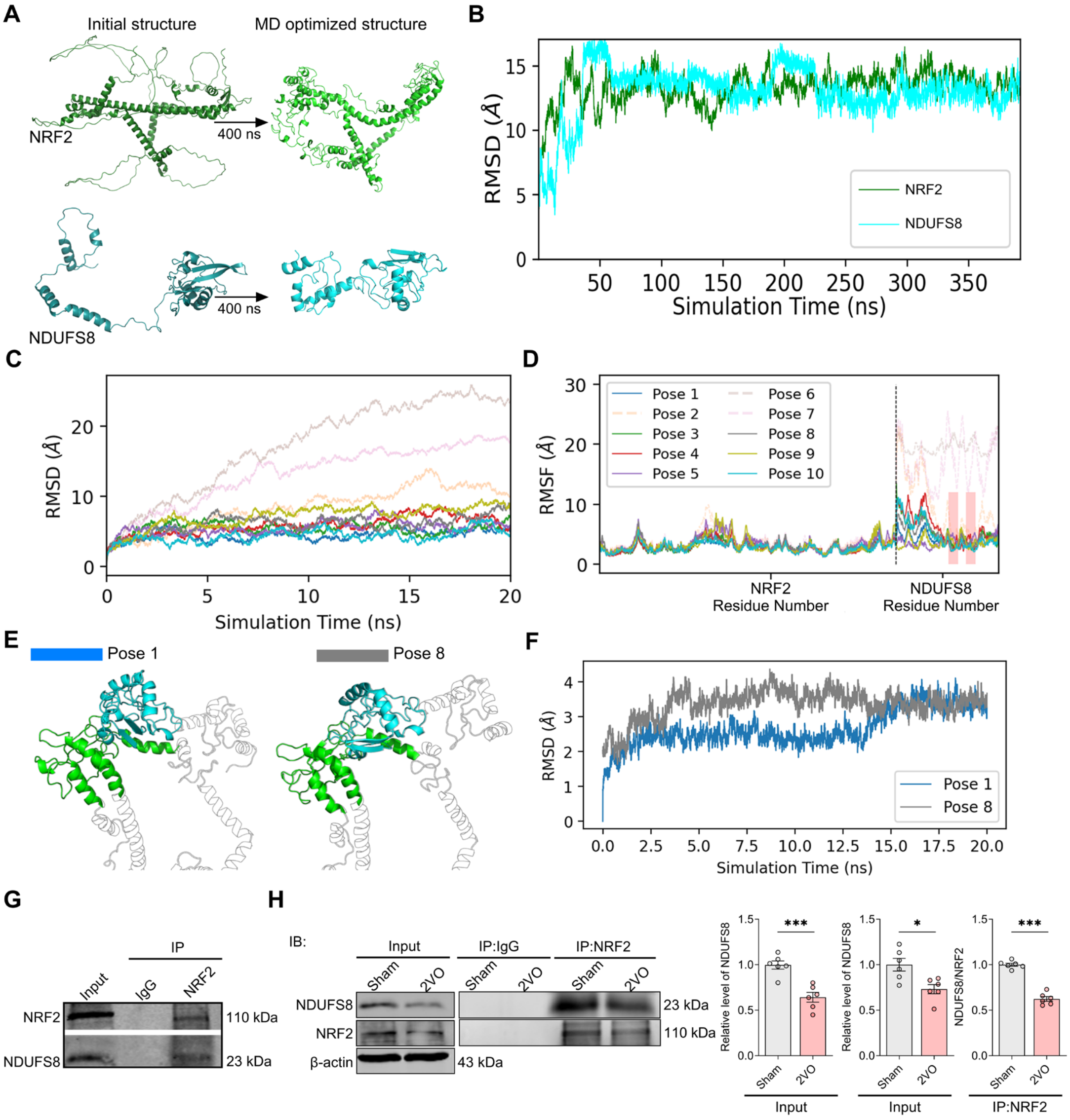

Figure S6. Comparison of two computational modellings of NRF2 and NDUFS8 binding. (A) 400 ns MD simulations to optimize the AlphaFold-predicted NRF2 and NDUFS8 structures. **(B)** The initial structure and the last frame of the MD trajectory are shown for comparison. RMSD was calculated against the first frame of the MD trajectory. **(C)** RMSD of protein backbone atoms during the 20 ns MD simulations of the NRF2-NDUFS8 poses. **(D)** RMSF of the protein non-hydrogen atoms. **(E)** The RMSD of intermolecular configurations for pose 1 and pose 8 during MD simulations. **(F)** The RMSD was calculated for the colored region in the upper panel and using the

first frame of pose 1 trajectory as reference. **(G)** Co-immunoprecipitation assay using NRF2 as bait protein demonstrated the interaction between NRF2 and NDUFS8. $n = 4$. **(H)** Reduced binding of NRF2 and NDUFS8 in the basal forebrain of 2VO rats. $n = 6$. For NDUFS8: Cohen's d = 2.91853; For NRF2: Cohen's d = 1.81679; For NDUFS8/NRF2: Cohen's d = 6.94473. Data are presented as the mean ± SEM. $*P < 0.05$, $***P < 0.001$. MD, molecular dynamics; RMSD, root mean squared deviations; RMSF, root mean squared fluctuations.

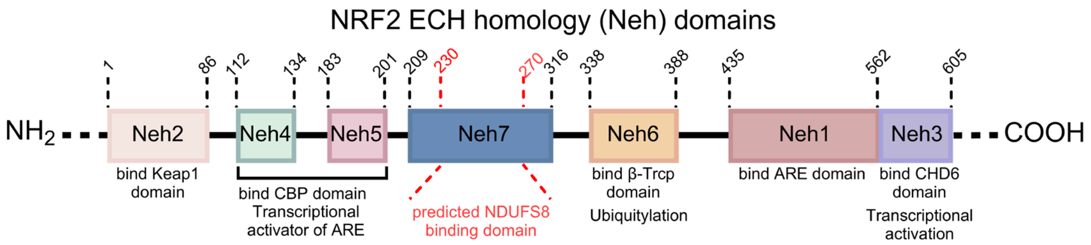

Figure S7. The function of NRF2-ECH homology (Neh1-7) domains. The structure of NRF2-ECH homology domains were described as previous study. Neh1 was the region binding to ARE domain of target gene to transcribe downstream genes; Neh2 and Neh6 domains were related to NRF2 degradation which related to Keap1 and ubiquitylation. Neh3, Neh4 and Neh5 participated in transcription of ARE genes as transcription activators. Neh7 was reported to suppress the NRF2/ARE pathway. In the present study, 230 ~ 270 residues in Neh7 domain predicted to bind NDUFS8 via protein-protein docking.

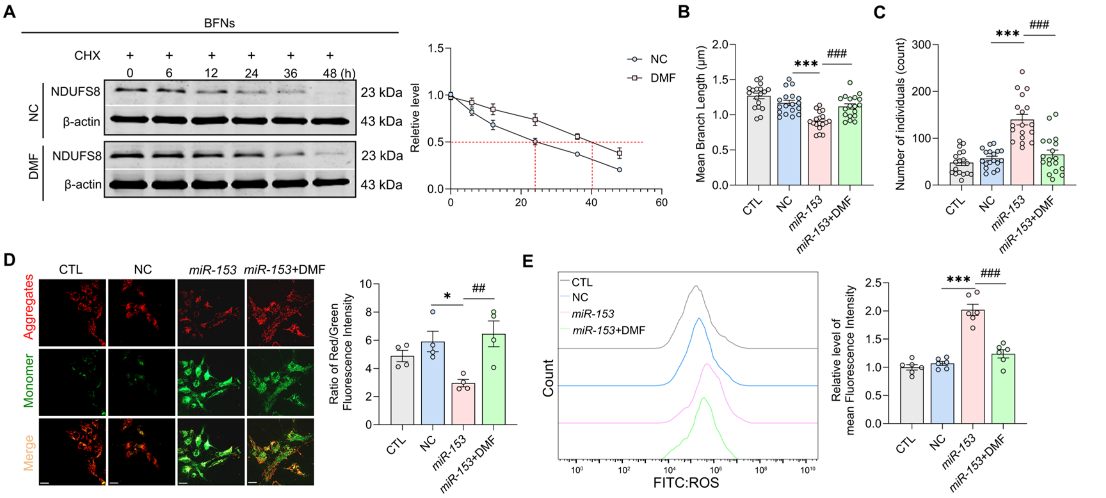

Figure S8. DMF increases NDUFS8 stability and improves mitochondrial function in BFNs. (A) The addition of DMF prolonged the half-life of the NDUFS8 protein in BFNs. $n = 8$. **(B-C)** DMF treatment increased mean branch length of mitochondria **(B)** and decreased mitochondrial individuals **(C)** in BFNs. $n = 18$ single mitochondria per group from 3 batches of cell culture. $\eta^2 = 0.43143$ **(B)** and 0.53517 **(C)**. **(D)** DMF treatment increased MMP detected with JC-1 signal. Scale bar = 20 μm. $n = 4$. $\eta^2 = 0.60048$. (**E**) DMF treatment reduced intracellular ROS level in BFNs. $n = 6$. $\eta^2 = 0.87425$. Data are presented as the mean ± SEM. $^{*}P < 0.05$, $^{***}P < 0.001$; $^{\#\#}P < 0.01$, $^{\#\#\#}P < 0.001$.

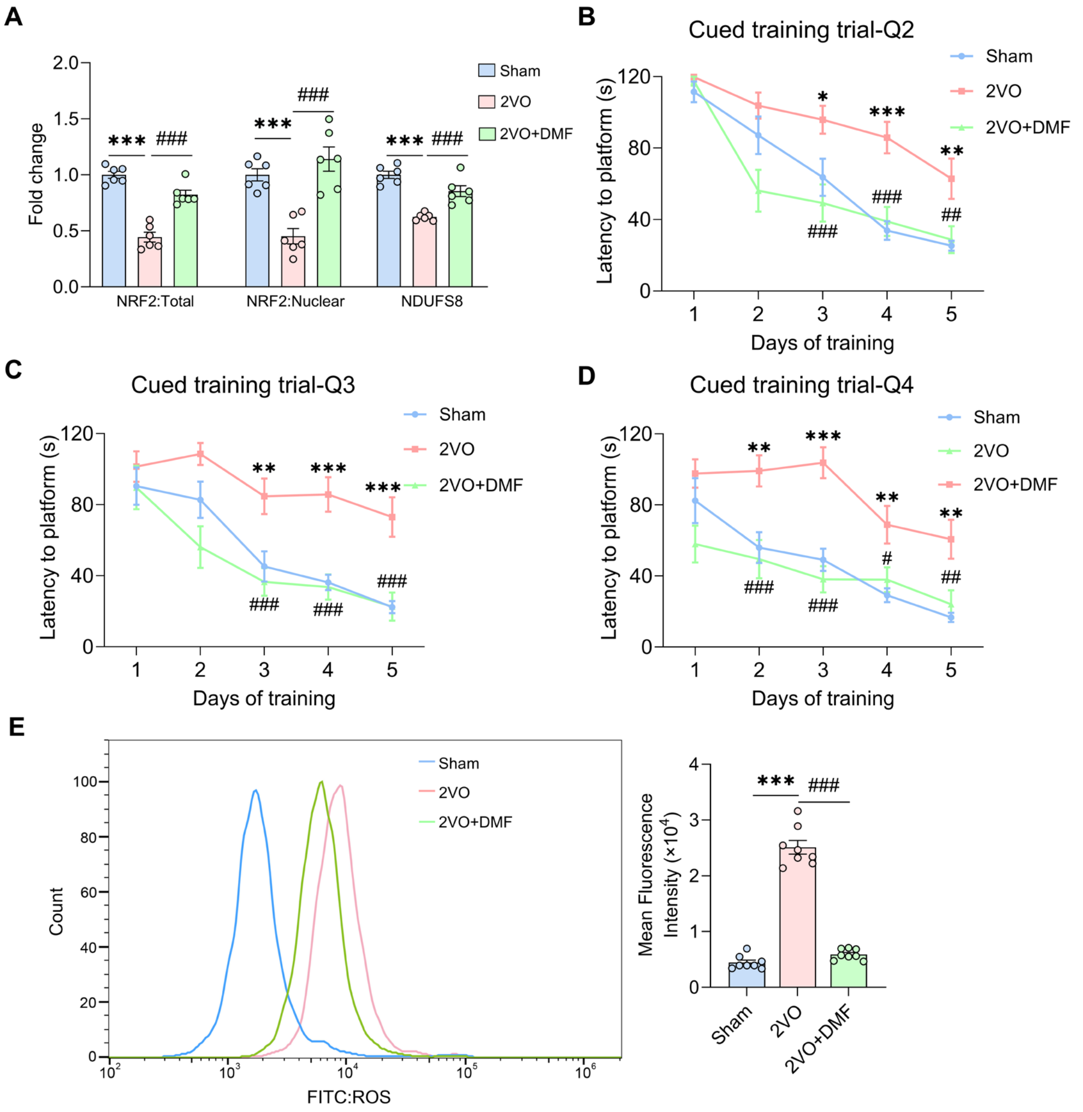

Figure S9. Gain-of-function of NRF2-NDUFS8 axis improves mitochondrial function and cognition of 2VO rats. (A) DMF treatment increased total or nuclear expression of NRF2 and expression of NDUFS8 in the basal forebrain of 2VO rats. n = 6. For total NRF2: $\eta^2 = 0.88110$; For nuclear NRF2: $\eta^2 = 0.73118$; For NDUFS8: $\eta^2 = 0.80039$. **(B-D)** DMF treatment decreased mean daily latency to locate the hidden platform (Q2/Q3/Q4). n = 14. $^{*}P < 0.05$, $^{**}P < 0.01$ $^{***}P < 0.001$ *vs.* Sham rats; $^{\#}P < 0.05$, $^{\#\#}P < 0.01$, $^{\#\#\#}P < 0.001$ *vs.* 2VO rats. **(E)** Addition of DMF decreased intracellular ROS level in the basal forebrain of 2VO rats. n = 6. $\eta^2 = 0.95390$. Data are presented

as the mean ± SEM. $^{***}P < 0.001$; $^{\#\#\#}P < 0.001$.

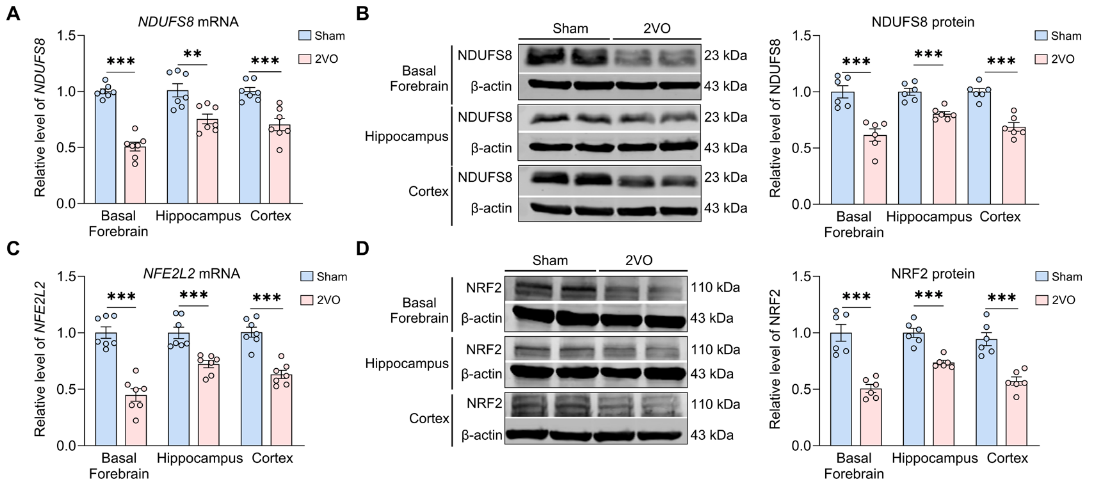

Figure S10. NRF2 and NDUFS8 are most significantly decreased in the basal forebrain rather than other regions. (A-B) The level of *NDUFS8* mRNA **(A)** and protein **(B)** were decreased more in the basal forebrain than in the hippocampus and cortex of 2VO rats. $n = 6 \sim 7$. For *NDUFS8* mRNA: Basal forebrain: Cohen's d = 5.91599; Hippocampus: Cohen's d = 1.86632; Cortex: Cohen's d = 2.51449; For NDUFS8 protein: Basal forebrain: Cohen's d = 2.87281; Hippocampus: Cohen's d = 2.96573; Cortex: Cohen's d = 3.70679. **(C-D)** The level of *Nfe2l2* mRNA **(C)** and protein **(D)** were decreased more in the basal forebrain than in the hippocampus and cortex of 2VO rats. $n = 6 \sim 7$. For *Nfe2l2* mRNA: Basal forebrain: Cohen's d = 3.94861; Hippocampus: Cohen's d = 2.46595; For Cortex: Cohen's d = 3.48899; For NRF2 protein: Basal forebrain: Cohen's d = 3.45131; Hippocampus: Cohen's d = 3.40418; Cortex: Cohen's d = 3.14346. Data are presented as the mean ± SEM. $^{**}P < 0.01$, $^{***}P < 0.001$.
